# “Influence of plasmids, selection markers and auxotrophic mutations on *Haloferax volcanii* cell shape plasticity”

**DOI:** 10.3389/fmicb.2023.1270665

**Published:** 2023-09-29

**Authors:** Megha Patro, Iain G. Duggin, Sonja-Verena Albers, Solenne Ithurbide

**Affiliations:** ^1^Molecular Biology of Archaea, Institute of Biology II, Faculty of Biology, University of Freiburg, Freiburg, Germany; ^2^Spemann Graduate School of Biology and Medicine, University of Freiburg, Freiburg, Germany; ^3^The Australian Institute for Microbiology and Infection, University of Technology Sydney, Sydney, NSW, Australia

**Keywords:** *Haloferax volcanii*, haloarchaea, cell shape, HDRB, plasmid, auxotrophy

## Abstract

*Haloferax volcanii* and other Haloarchaea can be pleomorphic, adopting different shapes, which vary with growth stages. Several studies have shown that *H. volcanii* cell shape is sensitive to various external factors including growth media and physical environment. In addition, several studies have noticed that the presence of a recombinant plasmid in the cells is also a factor impacting *H. volcanii* cell shape, notably by favoring the development of rods in early stages of growth. Here we investigated the reasons for this phenomenon by first studying the impact of auxotrophic mutations on cell shape in strains that are commonly used as genetic backgrounds for selection during strain engineering (namely: H26, H53, H77, H98, and H729) and secondly, by studying the effect of the presence of different plasmids containing selection markers on the cell shape of these strains. Our study showed that most of these auxotrophic strains have variation in cell shape parameters including length, aspect ratio, area and circularity and that the plasmid presence is impacting these parameters too. Our results indicated that Δ*hdrB* strains and *hdrB* selection markers have the most influence on *H. volcanii* cell shape, in addition to the sole presence of a plasmid. Finally, we discuss limitations in studying cell shape in *H. volcanii* and make recommendations based on our results for improving reproducibility of such studies.

## 1. Introduction

In the last couple of years, the field of cell biology of Archaea has developed immensely (van Wolferen et al., 2022). Amongst the current model organisms, *Haloferax volcanii* for which many tools have been developed has facilitated most of the cell biology discoveries in the Euryarchaeota phylum and helped build up the foundations of new paradigms in archaeal cell division, cell envelope biology and cell shape (Duggin et al., 2015; Pohlschroder and Schulze, 2019; Ithurbide et al., 2022). In general, how is cell shape established is an intriguing question in Haloarchaea as these species are commonly characterized as pleomorphic organisms that can harbor shapes ranging from rods and plates (also called disks or discoid) to triangles, squares, and more exotic forms (Mullakhanbhai and Larsen, 1975; Bisson-Filho et al., 2018; Walsh et al., 2019; Liao et al., 2021; Schwarzer et al., 2021; Tittes et al., 2021). In addition, several studies have shown that one species can adopt one shape or another depending on the growth phase. Indeed, in early log phase, rods can be observed in *H. volcanii* liquid cultures. Then, it transitions into a mixed population where rod cells coexist with plate cells as they grow in exponential phase to finally tend towards a homogenous population of plate cells of a smaller size by the time cells enter stationary phase (Li et al., 2019; de Silva et al., 2021). This growth phase dependent cell shape transitions have also been shown for *Haloferax gibbonsii*, *Haloarcula hispanica* and *Haloarchula californiae* (Schwarzer et al., 2021; Tittes et al., 2021). However, differences occur between the different species regarding the window of time when shape transitions occur, as well as the type of cell shape that are observed. Interestingly, another haloarchaeal species, *Halobacterium salinarum*, was shown to be mostly rod at all growth stages, with more than 90% of rods even in stationary phase (Eun et al., 2018). Furthermore, it has been shown that other external factors can influence cell shape including the media the cells are grown in and mechanical forces, further supporting the natural plasticity of haloarchaea cell shape (Bisson-Filho et al., 2018; Eun et al., 2018; de Silva et al., 2021).

Despite the interesting pleomorphism of Haloarchaea, little is known about the mechanisms involved in cell shape development and transition regulation. The archaeal specific tubulin CetZ1 was found to be essential for rod formation and knock out mutants remained in plate morphology (Duggin et al., 2015; de Silva et al., 2021). To the contrary, a mutant containing an insertion of a transposon upstream of HVO_2176 was found to maintain the rod shape and failed to transition to the plate morphology (Schiller et al., 2023). A comparative proteomics approach of the rod-only and plate-only mutants, and WT, in early log and in late log, identified several proteins implicated in cell shape development and transition (Schiller et al., 2023). These included HVO_2175, a SMC family homologue (Structural Maintenance of Chromosomes) called Sph3 whose disruption led the cells to only form rods, and its two neighboring genes *rdfA* (HVO_2174), important for rod formation, and *ddfA* (HVO_2176), important for plate formation. This study also identified “volactin”, a so-far uncharacterized homologue of actin, which appears to represent another group of cytoskeletal proteins alongside the tubulin family proteins, CetZ and FtsZ, important for the rod-to-plate cell shape transition (Schiller et al., 2023). Further studies are needed to determine the role of these genes in cell shape determination. Cell shape is a spatial cue for many core cell biology functions, including placement of the *H. volcanii* division plane (Walsh et al., 2019), the motility and chemotaxis machineries (Li et al., 2019; Nußbaum et al., 2020) and possibly many others.

Despite these advances, a recurring observation made by several laboratories might provide a limitation to consistency in future studies. Several studies have reported that morphological changes are induced in *H. volcanii* strains due to presence or absence of a plasmid based on the endogenous plasmid pHV2 (Supplementary Table S2). The first report of such a phenomenon was by Abdul Halim et al. (2016). While they were studying the phenotype of a deletion of the archaeosortase *artA* gene on cell morphology, they observed that during the first liquid culture inoculated from colonies on agar plates, about 20% of the cells were rods both in WT (H53Δ*trp*Δ*pyrE2*) and in Δ*artA* strains, whereas the same strains displayed 90% and 100% of rods, respectively, when harboring a plasmid (Abdul Halim et al., 2016). After a subsequent dilution of the liquid cultures, the Δ*artA* mutant only displayed plate cells whereas the same background strain bearing the plasmid (pTA963) were all rod shaped. It was also observed that the presence of pTA963 negatively impacted the growth rate of the Δ*artA* mutant compared to the strain without the plasmid. Corroborating these observations, de Silva et al., 2021 have shown that the early log rod development is much more visible for H26 (Δ*pyrE2*) or H98 (Δ*hdrB*, Δ*pyrE2*) containing the plasmid pTA962 compared to the plasmid-free strains in a rich liquid medium (de Silva et al., 2021).

Further potential complexities arise from this plasmid related cell shape variations in strains harboring mutations that affect cell morphology. For example, *ftsZ2* knockout strains show a very severe cell division defect and grow as very large plate cells whereas the same strain transformed with a plasmid is filamentous (Liao et al., 2021). The same is true for knock-down of *sepF*, another cell division protein (Nußbaum et al., 2021), whereas Δ*ftsZ1* cells do not become elongated in presence of the plasmid (Liao et al., 2021). These effects on cell shape, and their dependence on strain background and plasmid presence, potentially complicates the interpretation of the results of such genetic analyses.

To better understand how the plasmid used in these studies influences cell shape, here we characterized the cell shape of several *H. volcanii* strains that are commonly used as genetic backgrounds for selection during strain engineering namely: H26, H53, H77, H98, and H729 (Allers et al., 2004), and investigated the influence of the plasmid and various auxotrophic markers. Our results indicate that Δ*hdrB* marker strains and *hdrB* selection markers have the most influence on *H. volcanii* cell shape in addition to the sole presence of a plasmid. Finally, we discuss the current limitations in studying cell shape in *H. volcanii* and make recommendations based on our results for improving reproducibility of such studies.

## 2. Materials and methods

### 2.1. Strains and growth conditions

The list of strains and plasmids are available in Supplementary Table S1. *Haloferax volcanii* cells were grown in Hv-YPC or Hv-CA media.

Hv-YPC medium consisted of 0.5% (w/v) yeast extract (Oxoid 1447488-02), 0.1% (w/v) peptone (Oxoid 2145582), and 0.1% (w/v) casamino acids (Difco 0314259) dissolved in 18% buffered salt water (SW) 144 g/L NaCl (Roth 0962.3), 18 g/L MgCl_2_ * 6 H_2_O (Roth A537.1), 21 g/L MgSO_4_*7H_2_O (Roth P027.2), 4.2 g/L KCl (Roth P017.3), 12 mMTris/HCl (Roth 9090.2), pH 7.5, supplemented with 3 mM CaCl_2_ (Roth 5239.1)_,_ adjusted to a pH of 7.2 with KOH.

Hv-CA medium consisted of: 0.5% (w/v) casamino acids (Difco 0314259) dissolved in 18% SW, supplemented with 3 mM CaCl_2_ (Roth 5239.1), and 0.8 μg/mL of thiamine, and 0.1 μg/mL of biotin, adjusted to a pH of 7.2 with KOH.

For strains with auxotrophic mutations, the details of the culture conditions and additives to support growth in Hv-YPC or HV-CA are listed in Supplementary Table S1. The water used for preparing media was deionized water from a diH_2_O system. Solid agar media were prepared by addition of (1.5%) Bacto Agar (Difco 214010). Plates with strains were incubated at 45°C in a plastic container or plastic bag to limit evaporation.

Two methods were used for liquid cultures. For culture volume <5 mL, cells were grown in 15 mL glass tubes in a rotating platform and for cultures >5 mL, cells were grown in an Erlenmeyer flask covered with aluminum foil on a shaking platform. All cultures were grown at 45°C with a rotation of 110 rpm.

Transformation of *H. volcanii* via the Polyethylene glycol 600 (PEG600) method was performed as described before (Allers et al., 2004) and appropriate selection was used depending on the strain’s genetic background and the selection marker on the transforming plasmid. Non-methylated plasmids were extracted from *Escherichia coli dam^−^/dcm^−^* (C2925I, NEB) prior to transformation of *H. volcanii*. *E. coli* strains were cultured in LB medium, with the necessary antibiotics (100 μg/mL ampicillin, 30 μg/mL chloramphenicol, or 25 μg/mL kanamycin) and grown at 37°C under constant shaking.

### 2.2. Growth curves of *Haloferax volcanii* strains

Strains from glycerol stocks were streaked on solid agar medium plus auxotrophic requirements as needed, or, for strains with plasmids, on HV-CA agar plus required additives (see Supplementary Table S1), and then incubated at 45°C for 4–5 days to obtain isolated colonies. A single colony from plates was used to inoculate 5 mL of media on day 1. On day 2, the obtained culture was used to inoculate 50 mL of Hv-CA supplemented with appropriate additives (see Supplementary Table S1) so that the culture would reach an OD_600_ of 0.05 on the morning of day 3 and incubated at 45°C with shaking at 120 rpm. Measurement of optical density at 600 nm (OD_600_) was started on day 3 at an OD_600_ of 0.05 and manual sampling was performed up to 70 h.

### 2.3. Studies of *Haloferax volcanii* cell shape

#### 2.3.1. Growth conditions and sampling

Strains were streaked on Hv-YPC media for strains without a plasmid (see Supplementary Table S1) and appropriate Hv-CA media for strains with plasmids (see Supplementary Table S1) and grown for 4–5 days to obtain isolated colonies. A single colony from plate was inoculated in 5 mL of appropriate Hv-CA media for all the strains and grown overnight. The following day, the 5 mL culture was used to inoculate 15 mL of Hv-CA media in a 100 mL Erlenmeyer flasks with foil lid so that the culture would reach an OD_600_ of 0.01 the following morning. Samples were collected the following day at OD_600_ of 0.01, 0.03, 0.06, 0.1 and 0.2 and prepared for phase-contrast light microscopy.

#### 2.3.2. Phase-contrast light microscopy

A 5 μL culture sample was dropped onto an agarose pad [1% (w/v) agarose (Roth 3810.3), dissolved in 18% SW] and covered with a cover slip upon drying of the surface liquid. Micrographs were acquired on an inverted phase-contrast light microscope (Zeiss Axio Observer Z.1) at a magnification of 100X with an oil-immersion objective (Zeiss Plan-Apochromat 100×/1.40 NA Oil M27, with Immersol^™^ 518 F) and an exposure of 50 ms.

#### 2.3.3. Image and data analysis

Phase-contrast images were analyzed using Fiji combined with the MicrobeJ plugin (Schindelin et al., 2012; Ducret et al., 2016). Segmentation of archaeal cells was performed in MicrobeJ using the following settings: default mode for thresholding, scale 0.065 μm and area cutoff: 0.1—max and cell shape descriptors (circularity, length, area, and aspect ratio) were collected. For the analysis, cells which are present as aggregates or fragmented were manually discarded from the segmentation results.

For each strain and OD_600_, cell circularity was plotted as a frequency distribution obtained from the total number of cells analyzed (calculated and plotted in GraphPad Prism 6). The distribution was grouped into bins between 0 to 1 with increments of 0.1. Data were represented as frequency polygons and presented in a ridgeline plot to allow comparisons. Violin plots of the different cell shape parameters were created in GraphPad Prism 6, and plotted with the mean and standard deviation overlayed as a bar graph.

Categorization of cells into shape types rod (R), intermediate (I) and plates (P) was done in Excel with IF/AND functions based on the cell circularity features extracted from the segmentation/analysis in MicrobeJ and using the following parameters: a circularity between 1 and 0.8 included (closest to a circle) are corresponding to plate cells (P), values strictly in between 0.8 and 0.6 correspond to a mixed population of intermediate shapes (I) and values equal or below 0.6 (closest to an elongated shape) are rod cells (R). Percentages of each shape types from the total number of cells analyzed at a given OD_600_ were calculated in Excel and plotted as bar graphs.

Statistical analyses were performed in GraphPad Prism 6. If comparison was between two groups, an unpaired non-parametric Mann–Whitney test was carried out. For comparison between three or more groups, a non-parametric Kruskal–Wallis and Dunn’s multiple comparison tests were carried out. For the generation of the *p*-value heatmaps, *p*-values were calculated in python using SciPy (scipy.stats.kruskal) function and plotted as heatmaps with the seaborn package.

## 3. Results

### 3.1. Rod shape is transient during growth of wild-type *Haloferax volcanii* DS2

**Figure 1:**
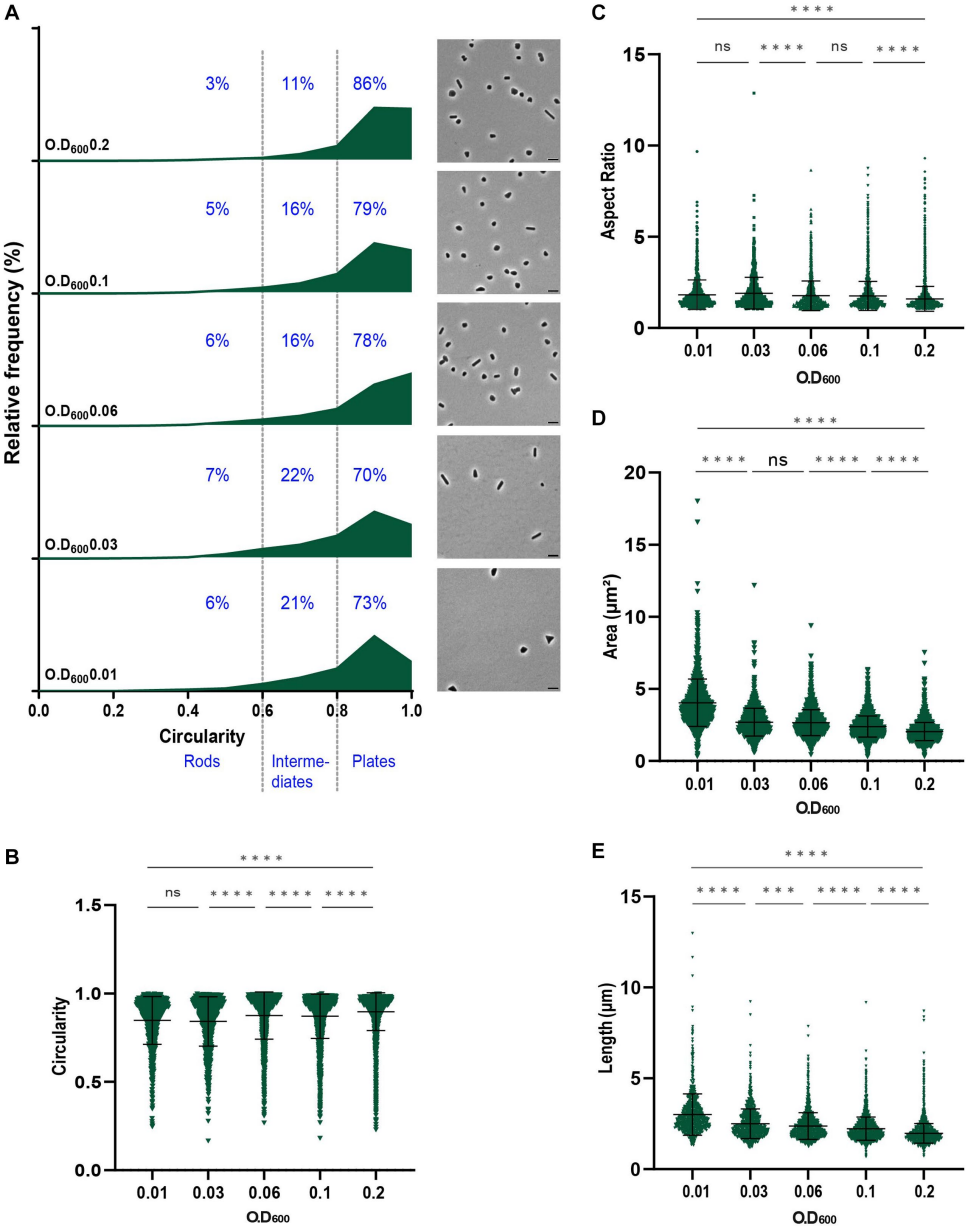
Analysis of the morphology of *H. volcanii* DS2 at different stages of growth. **(A)** Relative frequency distribution of cell circularity (left) measured from phase contrast image analysis (right) of DS2 samples collected at OD_600_ 0.01 to 0.2 (bottom to top). Cell types R (rods), I (intermediates) and P (plates) are determined depending on the cell circularity as described in the text and each cell shape percentage is indicated on the graphs. The *Y*-axis indicates the percentage of cells at each OD. Sum of the graph height per OD_600_ equals 100%. Scale bars on micrographs represent 4 μm. **(B)** Violin plot distribution of cells circularity at different OD_600._ Data set is the same as in **(A)**. **(C)** Violin plot distribution of cells aspect ratio at different OD_600_ **(D)** Violin plot distribution of cells area (μm^2^) at different OD_600_ **(E)** Violin plot distribution of cells length (μm) at different OD_600_. The statistical analysis in **(B–E)** were performed using Kruskal–Wallis-test in GraphPad Prism and data represent more than 1,100 cells from three independent experiments. Black line indicates mean; bottom and top lines indicate the standard deviation. Additional results of Kruskal–Wallis-tests are represented in Supplementary Figure S5.

Cell shape development during the growth phases of *H. volcanii* has only been reported for two of the commonly used auxotrophic strains, including H26 and H53 (Abdul Halim et al., 2016; Li et al., 2019; de Silva et al., 2021, Supplementary Table S2). To better characterize *H. volcanii* cell shape during growth in liquid medium and study the potential effects of the auxotrophic mutations on *H. volcanii* cell shape, we first studied the *H. volcanii* DS2 parental wild-type strain. Cells were grown in liquid Hv-CA medium, sampled at different stages of growth (OD_600_ 0.01, 0.03, 0.06, 0.1, and 0.2) and imaged by phase-contrast microscopy to obtain cell shape parameters, including cell length, aspect ratio, area, and circularity (Figure 1). Cell circularity was used as a proxy to characterize the degree of cell elongation and cells were categorized as plate cells (P, circularity 0.8–1), intermediate cells (I, circularity 0.6–0.8) or rod cells (R, circularity <0.6).

*H. volcanii* DS2 was pleomorphic throughout the different growth stages, showing a mixture of rods, intermediate and plate cells, with the majority of cells (~75%) existing as plates (Figure 1A). The proportion of plate cells increased as the culture progressed, with up to 86% of the population classified as plates at OD_600_ = 0.2 (Figure 1A). These data can also be seen as a significant increase of the mean of the circularity (Figure 1B) and a decrease of the mean of the aspect ratio (Figure 1C). This suggested that the formation of rods in early log phase, as described earlier in H26 and H53 (Li et al., 2019; de Silva et al., 2021), is also characteristic for the wild-type *H. volcanii* DS2.

Cell area (Figure 1D) and length (Figure 1E) measurements revealed a decrease in cell size during culture progression, that was also seen in each of the three shape classes (Supplementary Figure S1). Notably, rod cells and intermediate cells were significantly larger than plate cells at the same stages (Supplementary Figure S1). Intermediate cells generally had a size in between rod and plate cells, suggesting they may be in transition between plate and rod shapes.

### 3.2. Differing cell shape transitions in wild-type and auxotrophic *Haloferax volcanii* strains

Many of the common genetic tools of *H. volcanii* are based on selection via auxotrophic marker genes (Allers et al., 2004) and recombinant plasmids based on the small endogenous plasmid pHV2, which has been cured from wild-type DS2 (Wendoloski et al., 2001). Previous studies have also noticed some *H. volcanii* strains would form more rods with plasmid compared to those without plasmid. These observations, summarized in Supplementary Table S2, were made in the background strains H26 (Δ*pyrE2*), H53 (Δ*pyrE2*Δ*trpA*), and H98 (Δ*pyrE2*Δ*hdrB*) bearing plasmids based on pTA962/pTA963 derivatives containing the selection marker cassette p*_fdx_*-*pyrE2::hdrB* (Allers et al., 2004). Intrigued by the facts that (1) all the strains in which this phenomenon was observed share the Δ*pyrE2* mutation and that the strains H53 and H98 are derived from H26 and (2) that all the plasmids used were pTA962/963 derivatives with the same p*_fdx_*-*pyrE2::hdrB* selection cassette, we investigated whether the genetic background or the markers present on the plasmid could be responsible for the plasmid-dependent cell shape changes.

To test whether the auxotrophic mutations affect cell shape and may thereby be linked to the plasmid-mediated effects previously observed, we compared the cell shape development of five commonly used auxotrophic strains: H26 (Δ*pyrE2*), H53 (Δ*pyrE2*Δ*trpA*), H98 (Δ*pyrE2*Δ*hdrB*) as well as two other independent strains H77 (Δ*trpA*) and H729 (Δ*hdrB*) to wild-type DS2 (Supplementary Table S1).

**Figure 2:**
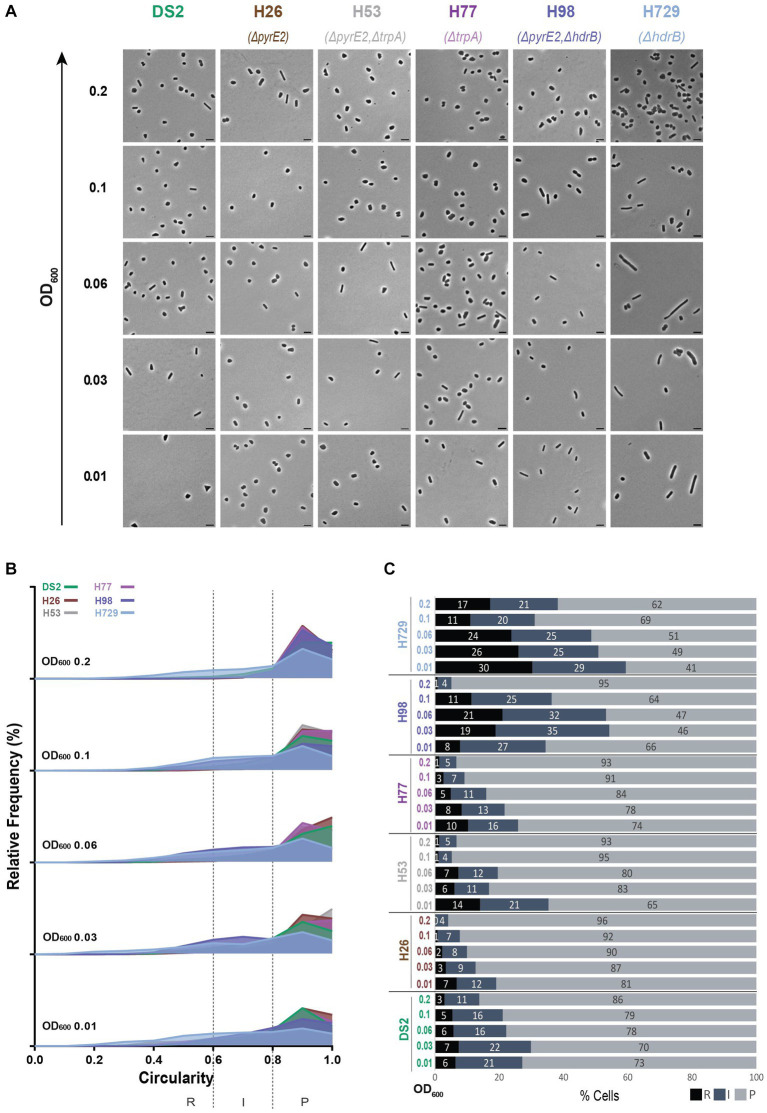
Morphological analysis of different auxotrophic *H. volcanii* strains at different stages of growth. **(A)** Phase contrast micrographs showing DS2, H26, H53, H77, H98 and H729 sampled at different growth stages from OD_600_ 0.01 (bottom) to 0.2 (top). Scale bars represent 4 μm. The genotype of each strain (coloured) is indicated at the bottom of the micrographs. **(B)** Relative frequency distribution of cell circularity measured from micrographs in **(A)** at OD_600_ 0.01 to 0.2 (bottom to top). Dashed vertical lines delimit the different cell types R (rods), I (intermediates) and P (plates) determined depending on the cell circularity as described in the text. The *Y*-axis indicates the percentage of cells at each OD. Sum of the graph height per OD_600_ equals 100%. **(C)** Bar graph indicating the percentage of each cell shape types at each sampled OD_600_ for each strain background. Colour representation—R (rods): black, I (intermediate): blue and P (plates): grey. **(B,C)** (*N* > 500 from three independent experiments). Data set for strain DS2 is the same as in Figure 1.

For the strains H26, H53 and H77, most cells were plates, representing more than 65% of the population at all stages of culture sampled similarly to DS2 (Figures 1, 2). The proportion of rods and intermediate cells decreased as the culture progressed into the exponential phase and cells transitioned to plate shape (~93% plates at OD_600_ = 0.2, Figures 2B,C). H26 showed the lowest proportion of rods and intermediate cells and this proportion decreased over time whereas most of the transition to plate cells occurred somewhat later, between OD_600_ = 0.06 and OD_600_ = 0.1, for H53 and H77.

Strains H98 and H729, however, showed a greater proportion of rods and intermediate cells compared to DS2, H26, H53, and H77 (Figure 2) and kept a ratio of around 50% rods and intermediate cells from OD_600_ = 0.01 to OD_600_ = 0.06 (Figures 2B,C). For H98, an abrupt transition to plate shape happened between OD_600_ = 0.1 to OD_600_ = 0.2 changing from 64% to 95% plates. However, H729 still displayed around 38% of rods and intermediate cells for 62% of plates at OD_600_ = 0.2. This might suggest that H729 is delayed in the transition to plate shape compared to H98 and to the other strains.

In addition to differences in the ratio between rods, intermediates and plates, our study showed differences of cell size between the strains and over time. Similar to DS2, the strains H53, H77, H729, and H98 showed the tendency to become smaller (see area Supplementary Figure S2C and length Supplementary Figure S2D) as the OD_600_ increased. However, H26 showed relatively small cells at OD_600_ = 0.01 and were larger by OD_600_ = 0.2 (see area Supplementary Figure S2C and longer Supplementary Figure S2D). The cells of H98, H53, H77, and H729 were overall larger than DS2 throughout, with H98 being the smallest of the four and H729 the largest (Supplementary Figures S2C,D). No substantive differences in the growth curves of these strains were observed (Supplementary Figure S3A). The H729 strain displayed the highest number of rods at all ODs (Figure 2C) and the highest aspect ratio and length (Supplementary Figures S2B,D) showing that this strain is also more elongated than the others; H729 rods cells sometimes appeared irregular and filamentous (Figure 2A). This phenotype will be discussed further below.

Overall, we observed that for all the strains tested, the general tendencies of *H. volcanii* cells are consistent with the previous studies of single strains: (1) the cells appear generally pleomorphic throughout the monitored growth period (early-mid log phase), (2) rods cells are more prevalent during the earlier stages of exponential growth in the conditions used, and (3) the cells of most strains become smaller as the OD increases. However, strain dependent features appear, including (1) the proportion of rods, intermediate and plate cells, (2) how long rod cells persist in the culture, (3) the size of the cells and (4) how elongated rods are.

### 3.3. Rod development is both plasmid and background strain dependent

#### 3.3.1. Cell shape in H26 backgrounds

We then analyzed the effect of various plasmids carrying different selection markers on the cell shape of the auxotrophic strains H26, H53, H98, H77, and H729.

H26 (Δ*pyrE2*) was transformed with pTA1392, a derivative from pTA962 containing the selection cassette p*_fdx_*-*pyrE2::hdrB* (Haque et al., 2020), or pTA230, containing the selection cassette p*_fdx_*-*pyrE2* (Allers et al., 2004).

**Figure 3:**
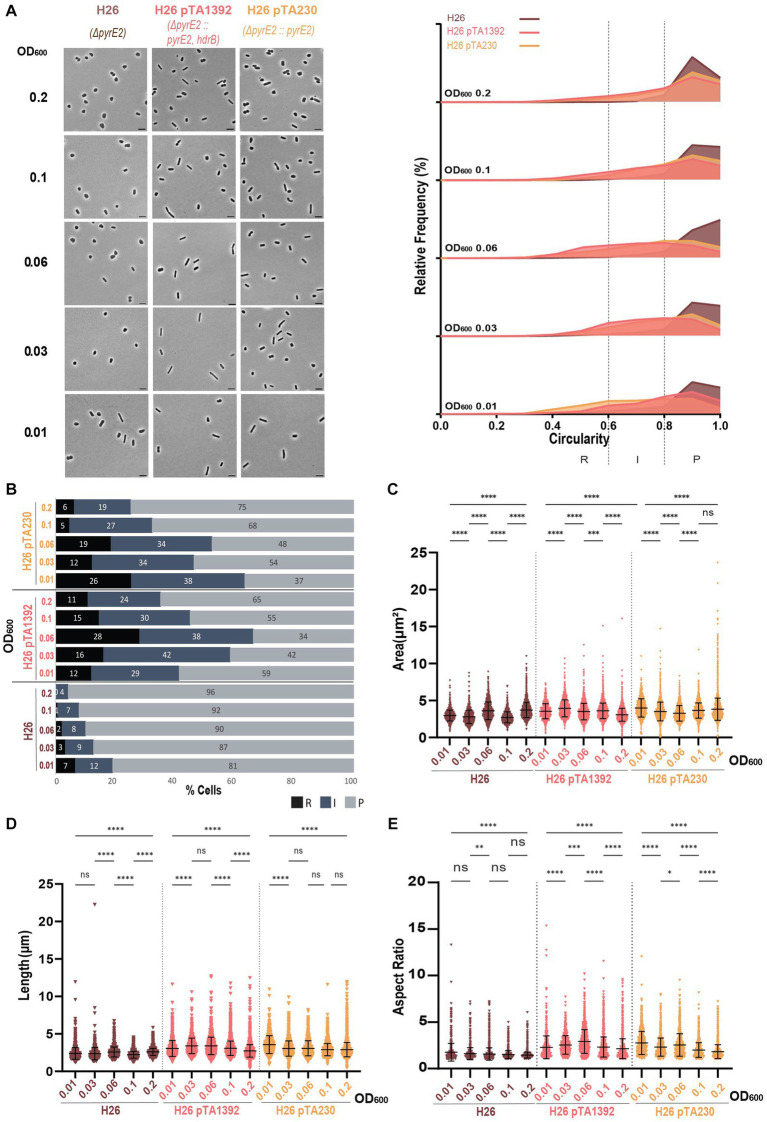
Morphological analysis of H26 with plasmids compared to H26 without plasmid at different stages of growth. **(A)** Left—phase contrast micrographs showing H26, H26 pTA1392, H26 pTA230 sampled at different growth stages from OD_600_ 0.01 (bottom) to 0.2 (top). Scale bars represent 4 μm. The genotype of each strain (coloured) is indicated at the bottom of the micrographs. Right—Relative frequency distribution of cell circularity measured from micrographs in **(A)** at OD_600_ 0.01 to 0.2 (bottom to top). Dashed vertical lines delimit the different cell types R (rods), I (intermediates) and P (plates) determined depending on the cell circularity as described in the text. The *Y*-axis indicates the percentage of cells at each OD. Sum of the graph height per OD_600_ equals 100%. **(B)** Bar graph indicating the percentage of each cell shape types at each sampled OD_600_ for each strain background. Colour representation—R (rods): black, I (intermediate): blue and P (plates): grey. **(C)** Violin plot distribution of cells area (μm^2^) at different OD_600_. **(D)** Violin plot distribution of cell length (μm) at different OD_600_. **(E)** Violin plot distribution of cells aspect ratio at different OD_600_. The statistical analysis in **(C–E)** were performed using Kruskal–Wallis-test in GraphPad Prism and data represent more than 1,300 cells from three independent experiments. Black line indicates mean; bottom and top lines indicate the standard deviation. Additional results of Kruskal–Wallis-tests are represented in Supplementary Figure S6. Data set for strain H26 is the same as in Figure 1.

As previously observed in H26 pTA962 (de Silva et al., 2021), the presence of pTA1392 or pTA230 resulted in a greater proportion of rod and intermediate cells during the early logarithmic phase compared to H26 without the plasmid (Figures 3A,B). No obvious rod-to-plate transition timing could be observed for H26 without plasmid, and rod shape was clear in H26 strains bearing a plasmid, which persisted to OD_600_ = 0.2 (Figures 3A,B).

The presence of the plasmid in the H26 background affected the proportions of the different cell shapes and the cell dimensions. While the H26 area increased overtime, the opposite happened for H26 pTA1392 and H26 pTA230 (Figure 3C) which is more similar to what happened in DS2 (Figure 1D) and the other backgrounds strains studied in Supplementary Figure S2C. The two plasmid-bearing strains were overall more elongated than H26 without plasmid (Figures 3D,E) and showed lower circularity (Supplementary Figure S4A). H26 pTA1392 was marginally the most elongated of the three strains (Figures 3D,E). Note that H26 pTA230 and H26 pTA1392 were both grown in Hv-CA without additional uracil as the p*_fdx_*-*pyrE2* marker was present on the plasmids. The main difference between these two conditions is the additional presence of the *hdrB* marker expressed from p*_fdx_* on pTA1392. This increases the gene copy number of the *hdrB* gene that is present both in the chromosome and the plasmid, expected to cause overexpression of the gene. These results would suggest that the additional expression of *hdrB* differentially impacts *H. volcanii* cell shape.

#### 3.3.2. Cell shape in H53 and H77 backgrounds

**Figure 4:**
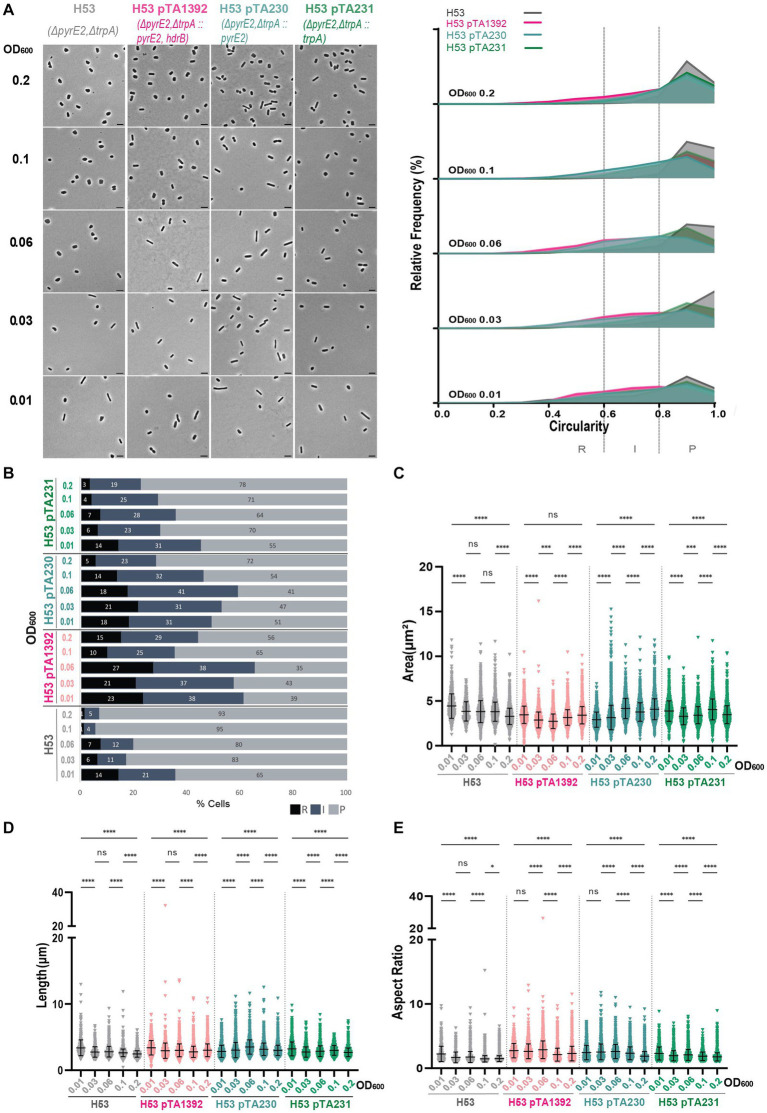
Morphological analysis of H53 with plasmids compared to H53 without plasmid at different stages of growth. **(A)** Left—phase contrast micrographs showing H53, H53 pTA1392, H53 pTA230, H53 pTA231 sampled at different growth stages from OD_600_ 0.01 (bottom) to 0.2 (top). Scale bars represent 4 μm. The genotype of each strain (coloured) is indicated at the bottom of the micrographs. Right—relative frequency distribution of cell circularity measured from micrographs in **(A)** at OD_600_ 0.01 to 0.2 (bottom to top). Dashed vertical lines delimit the different cell types R (rods), I (intermediates) and P (plates) determined depending on the cell circularity as described in the text. The *Y*-axis indicates the percentage of cells at each OD. Sum of the graph height per OD_600_ equals 100%. **(B)** Bar graph indicating the percentage of each cell shape types at each sampled OD_600_ for each strain background. Colour representation—R (rods): black, I (intermediate): blue and P (plates): grey. **(C)** Violin plot distribution of cells area (μm^2^) at different OD_600_. **(D)** Violin plot distribution of cell length (μm) at different OD_600_. **(E)** Violin plot distribution of cells aspect ratio at different OD_600_. The statistical analysis in **(C–E)** were performed using Kruskal–Wallis-test in GraphPad Prism and data represent more than 500 cells from three independent experiments. Black line indicates mean; bottom and top lines indicate the standard deviation. Additional results of Kruskal–Wallis-tests are represented in Supplementary Figure S7. Data set for strain H53 is the same as in Figure 1.

We complemented H53 (Δ*pyrE2*Δ*trpA*) with pTA230 (p*_fdx_*-*pyrE2*), pTA231 (p*_fdx_*-*trpA*) and pTA1392 (p*_fdx_*-*pyrE2::hdrB*), similar to the plasmids used in previous works (Abdul Halim et al., 2016; de Silva et al., 2021, Supplementary Table S2), and H77 (Δ*trpA*) was complemented with pTA231 (p*_fdx_*-*trpA*). In the H53 background, the plasmids resulted in a higher proportion of rods and intermediate cells, noticeable particularly with pTA1392 (Figures 4A,B; Supplementary Figure S4B). For H53 pTA1392 and H53 pTA230, the proportion of rods and intermediate cells increased until OD_600_ = 0.06 to reach a peak of 65% and 59%. In between OD_600_ = 0.06 and OD_600_ = 0.1 the population cell shape transition occurred, and the proportion of cell shapes reversed in favor of plate cells. However, for H53 pTA231 the peak of rods and intermediates happened early on at OD_600_ = 0.01 and a rather smooth decrease of the proportion of rod cells was visible up to OD_600_ = 0.2.

Interestingly, H53 pTA1392 cells were generally smaller than the cells of the other strains (see area and length Figures 4C,D) but their aspect ratio was relatively high (Figure 4E) suggesting that in H53, pTA1392 results in greater elongation and thinning of the cells. The similarity to the results for H26 pTA1392 would suggest a similar effect of the additional *hdrB* marker on the cell morphology in strains deleted for *pyrE2*.

**Figure 5:**
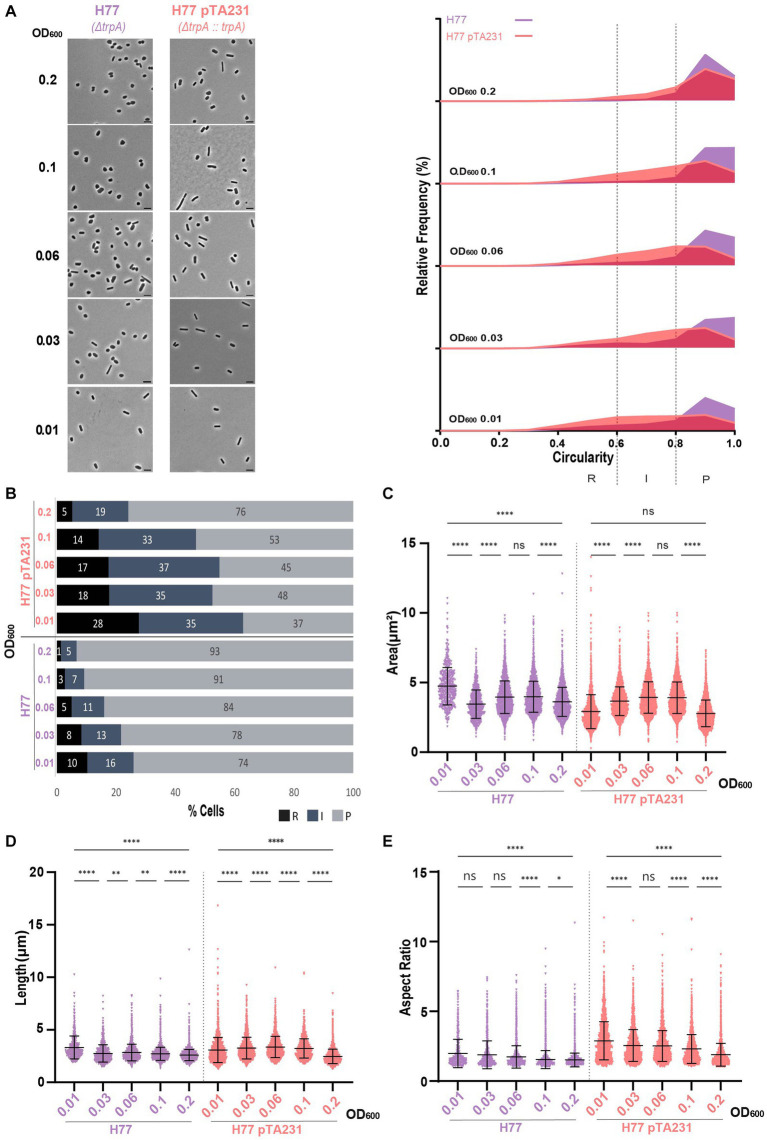
Morphological analysis of H77 with plasmids compared to H77 without plasmid at different stages of growth. **(A)** Left—phase contrast micrographs showing H77, H77 pTA231 sampled at different growth stages from OD_600_ 0.01 (bottom) to 0.2 (top). Scale bars represent 4 μm. The genotype of each strain (coloured) is indicated at the bottom of the micrographs. Right—relative frequency distribution of cell circularity measured from micrographs in **(A)** at OD_600_ 0.01 to 0.2 (bottom to top). Dashed vertical lines delimit the different cell types R (rods), I (intermediates) and P (plates) determined depending on the cell circularity as described in the text. The *Y*-axis indicates the percentage of cells at each OD. Sum of the graph height per OD_600_ equals 100%. **(B)** Bar graph indicating the percentage of each cell shape types at each sampled OD_600_ for each strain background. Colour representation—R (rods): black, I (intermediate): blue and P (plates): grey. **(C)** Violin plot distribution of cells area (μm^2^) at different OD_600_. **(D)** Violin plot distribution of cells length (μm) at different OD_600_. **(E)** Violin plot distribution of cells aspect ratio at different OD_600_. The statistical analysis in **(C–E)** were performed using Kruskal–Wallis-test in GraphPad Prism and data represent more than 500 cells from three independent experiments. Black line indicates mean; bottom and top lines indicate the standard deviation. Additional results of Kruskal–Wallis-tests are represented in Supplementary Figure S8. Data set for strain H77 is the same as in Figure 1.

The presence of pTA231 in H77 induced a relatively high proportion of rods and intermediate cells starting from 63% at OD_600_ = 0.01 and the transition towards a majority of plate cells occurred in between OD_600_ = 0.06 and OD_600_ = 0.1 (Figures 5A,B; Supplementary Figure S4C). Interestingly in the case of the H77 background, the presence of the plasmid did not increase the cell area (Figure 5C). However, cells bearing pTA231 are generally longer (Figure 5D) and have a higher aspect ratio than H77 without plasmid (Figure 5E) suggesting that in this strain, the presence of the plasmid pTA231 induces a remodeling of the cell towards elongated rods.

#### 3.3.3. Cell shape in H729 and H98 backgrounds

The same experiments were conducted for H98 (Δ*pyrE2*Δ*hdrB*) transformed with pTA1392 (p*_fdx_*-*pyrE2::hdrB*), pTA230 (p*_fdx_*-*pyrE2*) and pTA233 (p*_fdx_*-*hdrB*), and H729 (Δ*hdrB*) transformed with pTA1392 and pTA233.

**Figure 6:**
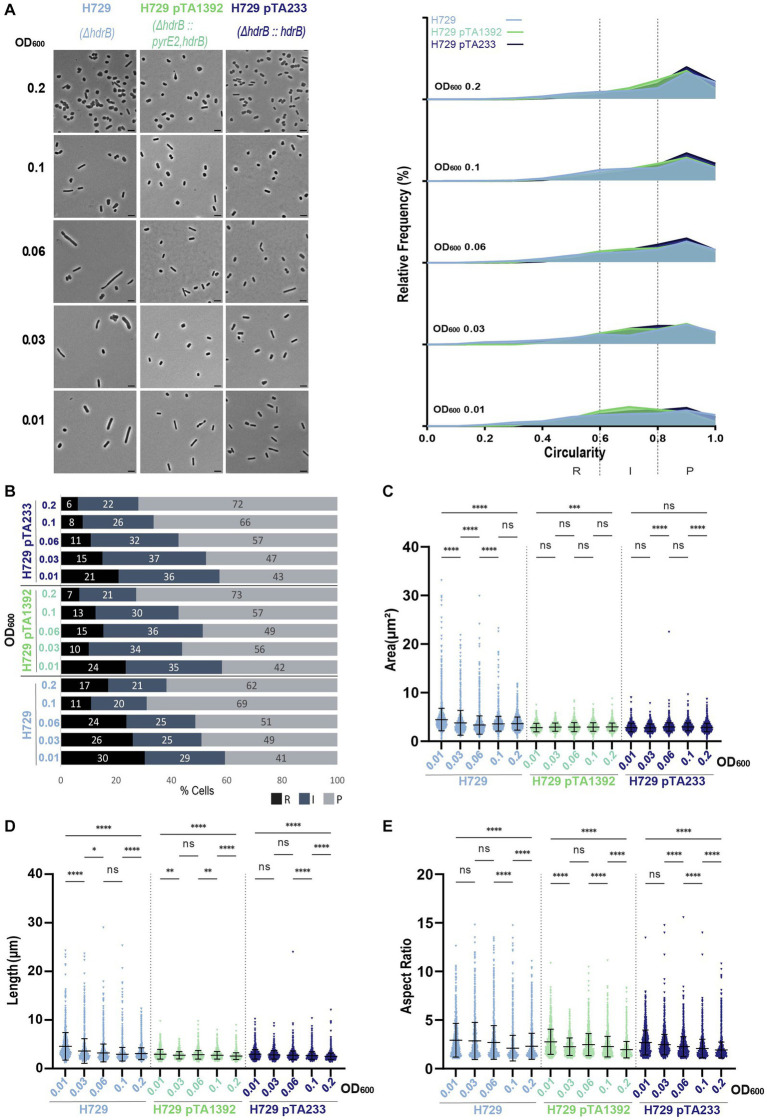
Morphological analysis of H729 with plasmids compared to H729 without plasmid at different stages of growth. **(A)** Left—phase contrast micrographs showing H729, H729 pTA1392, H729 pTA233 sampled at different growth stages from OD_600_ 0.01 (bottom) to 0.2 (top). Scale bars represent 4 μm. The genotype of each strain (coloured) is indicated at the bottom of the micrographs. Right—relative frequency distribution of cell circularity measured from micrographs in **(A)** at OD_600_ 0.01 to 0.2 (bottom to top). Dashed vertical lines delimit the different cell types R (rods), I (intermediates) and P (plates) determined depending on the cell circularity as described in the text. The *Y*-axis indicates the percentage of cells at each OD. Sum of the graph height per OD_600_ equals 100%. **(B)** Bar graph indicating the percentage of each cell shape types at each sampled OD_600_ for each strain background. Colour representation—R (rods): black, I (intermediate): blue and P (plates): grey. **(C)** Violin plot distribution of cells area (μm^2^) at different OD_600_. **(D)** Violin plot distribution of cell length (μm) at different OD_600_. **(E)** Violin plot distribution of cells aspect ratio at different OD_600_. The statistical analysis in **(C–E)** were performed using Kruskal–Wallis-test in GraphPad Prism and data represent more than 900 cells from three independent experiments. Black line indicates mean; bottom and top lines indicate the standard deviation. Additional results of Kruskal–Wallis-tests are represented in Supplementary Figure S9. Data set for strain H729 is the same as in Figure 1.

For H729, which had the largest cells compared to the other strains without plasmid and showed the highest proportion of rods and intermediate cells (Figure 2; Supplementary Figure S2), the presence of the plasmids pTA1392 or pTA233 did not increase the percentage of rods and intermediate cells (Figures 6A,B). To the contrary, at OD_600_ = 0.2, H729 pTA1392 and H729 pTA233 showed less rods than H729 without plasmid. Additionally, the cells harboring pTA1392 and pTA233 were globally smaller, shorter, less elongated and more circular than H729, pTA233 conferring the smaller size and highest circularity (Figures 6C–E; Supplementary Figure S4E).

The difference between H729 without plasmid and with plasmid, might suggest a fundamental difference between the addition of thymidine and hypoxanthine in the media and the expression of *hdrB* from the plasmid for supporting H729 cell development in Hv-CA. However, it is interesting to note that differences could be observed between H729 pTA1392 and H729 pTA233 in terms of cell size and growth despite their same growth medium (Figure 6; Supplementary Figure S3F). These differences might be due to the extra copy of *pyrE2* expressed from pTA1392 or that *hdrB* is transcribed from the p*_fdx_*-*pyrE2::hdrB* cassette in a multicistronic mRNA and that the expression of *hdrB* in that context is most likely different than for p*_fdx_*-*hdrB* from pTA233.

**Figure 7:**
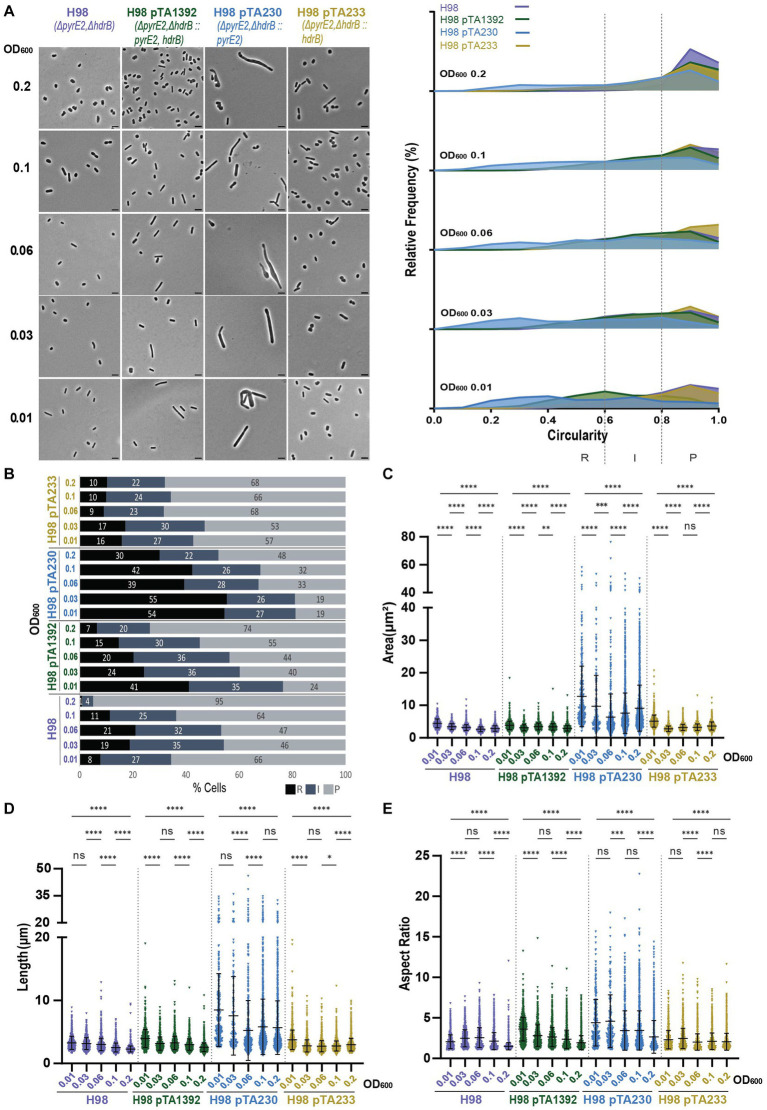
Morphological analysis of H98 with plasmids compared to H98 without plasmid at different stages of growth. **(A)** Left—phase contrast micrographs showing H98, H98 pTA1392, H98 pTA230, H98 pTA233 sampled at different growth stages from OD_600_ 0.01 (bottom) to 0.2 (top). Scale bars represent 4 μm. The genotype of each strain (coloured) is indicated at the bottom of the micrographs. Right—relative frequency distribution of cell circularity measured from micrographs in **(A)** at OD_600_ 0.01 to 0.2 (bottom to top). Dashed vertical lines delimit the different cell types R (rods), I (intermediates) and P (plates) determined depending on the cell circularity as described in the text. The *Y*-axis indicates the percentage of cells at each OD. Sum of the graph height per OD_600_ equals 100%. **(B)** Bar graph indicating the percentage of each cell shape types at each sampled OD_600_ for each strain background. Colour representation—R (rods): black, I (intermediate): blue and P (plates): grey. **(C)** Violin plot distribution of cells area (μm^2^) at different OD_600_. **(D)** Violin plot distribution of cell length (μm) at different OD_600_. **(E)** Violin plot distribution of cells aspect ratio at different OD_600_. The statistical analysis in **(C–E)** were performed using Kruskal–Wallis-test in GraphPad Prism and data represent more than 1,700 cells from three independent experiments. Black line indicates mean; bottom and top lines indicate the standard deviation. Additional results of Kruskal–Wallis-tests are represented in Supplementary Figure S10. Data set for strain H98 is the same as in Figure 1.

Interestingly, H98 pTA233 grown in Hv-CA + uracil also showed cells that were smaller, shorter, and more circular compared to H98 or H98 pTA1392 (Figures 7C,D; Supplementary Figure S4D). This further supports the idea that an external addition of 40 μg/mL of thymidine and hypoxanthine, or the expression of *hdrB* from p*_fdx_*-*pyrE2::hdrb* or p*_fdx_*-*hdrb* does not restore the cell’s prototrophy to the same extent and differently affects the growth and the cell shape development in Δ*hdrB* strains as compared to the wildtype (Figure 7; Supplementary Figures S3, S4D).

The presence of pTA233 in H98 did not drastically increase the percentage of rods and intermediate cells compared to H98 without plasmid but seemed to help maintain a rather stable proportion of the different cell shapes overtime. Indeed, H98 transitioned to plate shape between OD_600_ = 0.1 and OD_600_ = 0.2 to attain 95% of plates whereas H98 pTA233 still showed 33% of rods and intermediates at OD_600_ = 0.2 (Figures 7A,B).

Both pTA1392 and pTA230 increased the percentage of rods and intermediate cells in H98 but in different manners (Figures 7A,B). While the percentage of rods and intermediate cells was largely increased compared to H98 at OD_600_ = 0.01 for both strains, this percentage gradually decreased overtime for H98 pTA1392 down to 27% at OD_600_ = 0.2 (transition between OD_600_ = 0.1 and OD_600_ = 0.2) whereas it stayed at high levels for H98 pTA230 (52%). Indeed, while pTA1392 reduced the overall size of the cells compared to H98, the presence of pTA230 greatly increased the size (area and length, Figures 7C,D) and cells were much more elongated (see aspect ratio and circularity, Figure 7E; Supplementary Figure S4D) than all the other H98 background strains. The genotypes and phenotypes of H98 pTA230 (Δ*pyrE2*Δ*hdrB/*p*_fdx_*-*pyrE2*) resembled the one of H729 (Δ*hdrB*), in the sense that the *pyrE2* deletion is compensated by the plasmidic expression of p*_fdx_*-*pyrE2* and addition of thymidine and hypoxanthine is required for growth in Hv-CA. In both cases, the cells were larger and formed irregular and elongated rods (Figures 7A,C–E). This phenotype might reflect the cumulative effect of the incomplete support of cell morphology development when thymidine and hypoxanthine are provided in the media and the presence of a plasmid.

## 4. Discussion

This study reports an analysis of the cell shape of the *H. volcanii* DS2 strain during the different stages of the exponential growth and could show that the rod development and cell shape transition is a natural behavior of *H. volcanii*. As previously seen in other genetic backgrounds, the development of rods is restricted to the early stages of exponential growth (Abdul Halim et al., 2016; Li et al., 2019; de Silva et al., 2021). However, the percentage of rods is relatively small for DS2 in the tested conditions. The analysis of the various cell shape parameters clearly showed that the cells become smaller as the culture goes through the exponential phase as seen earlier for other *Haloarchaea* (Schwarzer et al., 2021). A phenomenon that has been observed for batch cultures of bacteria too. Indeed, the cell mass as well as the growth rate of *E. coli* K-12 strain MG1655 cultivated in batch cultures in Luria-Bertani broth drop considerably from OD_600_ = 0.3 and keep decreasing gradually until stationary phase (Sezonov et al., 2007) and cell length has been shown to decrease over time too (Shi et al., 2021). It was shown that this reduction of cell mass was concomitant with the impoverishment of utilizable carbon source in the media (Sezonov et al., 2007). While it has been shown that *E. coli* surface area/volume varies through time, the mechanisms that govern the control of cell width, length, volume and surface area are still debated (Shi et al., 2017; Colavin et al., 2018; Oldewurtel et al., 2021). Apart from nutrient availability, DNA replication could affect cell size and shape as observed in bacteria (Westfall and Levin, 2017). *H. volcanii* has a multipartite and multicopy genome with multiple active origins of replication (for review Pérez-Arnaiz et al., 2020). So far the existence of a clear cell cycle in *H. volcanii* and the coupling of DNA replication, cell growth, cell division and chromosome segregation mechanisms are unknown. However, it is known that the genome copy number decreases between exponential phase and stationary phase from around 18 copies to around 10 copies (Breuert et al., 2006) so a possible link between the reduction of genome copy number and the reduction of cell size would be worth studying.

Additionally to a diminution of the population cell size over time, our study showed that the rod cells and intermediate cells in low OD cultures are also larger (larger area) than plate cells. It would be interesting to study how biomass is accumulated in the different cell shapes. *H. volcanii* cell envelope is constituted of an S-layer Glycoprotein and has been proposed to be newly incorporated at the division site (Abdul-Halim et al., 2020). However, nothing is known about how growth and S-layer incorporation are regulated, nor how this is coordinated with cell shape determination. A recent study in another haloarchaeon *Halobacterium salinarum* demonstrated that this species grows following the adder model where cells grow by adding a constant volume between two cell cycles (Eun et al., 2018). However, this study was conducted in conditions where all the cells were maintained in rod shape so there is currently no idea whether plate cells are growing the same way.

Another yet challenging side of *H. volcanii* cell shape plasticity is that the presence of a plasmid affects the cell shape of *H. volcanii* whether it is by supporting the development of rods (de Silva et al., 2021) or changing the morphological phenotypes of mutant strains (Abdul Halim et al., 2016; Liao et al., 2021; Nußbaum et al., 2021). Such effects may complicate studies of cell division, cell growth, cell morphology and cell motility where cell shape is an important component of the mechanisms studied. Based on this we sought to conduct a comparative study of the cell shape of the most commonly used background strains without and with a transformed plasmid in order to either find conditions where this would not happen or provide guidance for improving the current system. While we are aware that some limitations apply to our study, like for example the fact that each strain was cultivated in different media, i.e., Hv-CA supplemented adequately depending on the strains’ auxotrophic state, and that direct comparison of them is somewhat questionable or the fact that we did not compare with the strain DS70 depleted from the plasmid pHV2 (Wendoloski et al., 2001) from which H26, H53, H77, H729, and H98 are derived from (Allers et al., 2004), we were able to highlight several patterns reflecting the presence or absence of a plasmid on *H. volcanii* cell shape. Most commonly, the plasmid increases the percentages of rods and intermediate cells compared to strains without plasmid. Sometimes it rather affects the dimensions of the cells (area, aspect ratio, length) (example H729) rather than changing the percentage of the different cell shapes. Other times it would change the timing during rods are developed and most of the times it is a combination of several of these phenomena. These phenotypes are most likely due to a combinatorial effect of the plasmid presence and of the different auxotrophic deletions and markers used for selection. Indeed, we have shown that the deletion of the various auxotrophic markers does have an impact on the cell shape, whether it is by increasing the percentage of rods and intermediate cells or/ and changing the cell dimensions independently of the presence of a plasmid. For example, the Δ*trpA* deletion (present in H53 and H77) seems to increase the cell size but does not change the relative proportion of the cell types nor the timing of development of the rods compared to DS2.

The most striking impact on cell shape observed in this study was for the Δ*hdrB* deletion. Indeed, the deletion of *hdrB* itself induces a greater proportion of elongated cells, as seen both in H729 and H98 backgrounds. The cells are not only more elongated but also larger than DS2 especially for H729. A phenotype that could be partially reversed when the *hdrb* gene was provided on a plasmid. It shows that the external addition of thymidine and hypoxanthine required for growth of *hdrb* backgrounds in Hv-CA is not sufficient to support a proper cell shape (i.e., cell size and shape closer to DS2). The supplementation of thymidine and hypoxanthine to the medium is enough for the cells to grow (Supplementary Figure S3F) but most likely not sufficient to support normal cellular morphology thus cells tend to grow larger and more elongated and irregular as compared to DS2 for example. To the opposite, the complementation of *hdrB* by the plasmidic copy might better restore the prototrophy through endogenous biosynthesis that may be more efficient than uptake from the medium, and therefore most likely better restore the cell physiology. However, the complementation of the strains with plasmid carrying *hdrb* did not reduce the proportion of rods as one could have expected but to the opposite, it increased further the proportion of rods and intermediate cells in the *hdrb* background suggesting a combinatorial effect of the plasmid presence and the auxotrophy on cell shape in this background. Remarkably, for the H26 (Δ*pyrE2*) and H53 (Δ*pyrE2*Δ*trpA*) strains that became merodiploids for *hdrb* by the transformation of pTA1392 (p*_fdx_*-*pyrE2::hdrB*), a greater proportion of rods and intermediate cells could be observed compared to the same strains transformed by other plasmids without the *hdrB* marker. Suggesting that in this case, the cumulative expression of the chromosomal and plasmid copies of *hdrB* does induce rod formation even more than other plasmids in these backgrounds. This suggests that the over expression of the *hdrb* gene severely induces the formation of rods additionally to the sole effect of the plasmid presence. This could also suggest that the expression of *hdrb* from the p*_fdx_* promoter on the plasmid could be too high as a stimulation of rod formation is observed too in strains H98 and H729 transformed with a plasmid. By extrapolation, we could hypothesize that the increase of rods percentage observed for the Δ*hdrB* strains grown in Hv-CA could be attributed to an over activation of the *hdrB* downstream pathways by addition of thymidine and hypoxanthine rather suggesting that the concentration of the additives could be too high.

*Hdrb* encodes for a dihydrofolate reductase involved in the tetrahydrofolate biosynthesis which in turn participates in many reactions for the synthesis of major cellular components such as methionine, purine, pyrimidine and glycine. Such enzymes are found universally and often essential to cells, which makes its activity a common target for various anti-cancerous and antibacterial treatments (Hawser et al., 2006; Chawla et al., 2021). In *H. volcanii*, the deletion of *hdrb* is not lethal but causes a thymidine auxotrophy and deletion mutants require the addition of thymidine and hypoxanthine to grow in Hv-CA (Ortenberg et al., 2000; Falb et al., 2008). In bacteria, the level of thymidine has been shown to greatly affect the cells leading eventually to death under thymidine starvation conditions (Ahmad et al., 1998). It has also been shown in *E. coli* that both an under- and oversupply of thymidine in a Thy^−^ affect the activity of the ribonucleoside diphosphate reductase (RNR) which in turn unbalances the pools of dNTPs and affect the replication velocity (Molina et al., 1998; Zhu et al., 2017). Additionally, a lower titration of RNR did increase the size of the cells by three times and some strains were shown to form filament under thymidine starvation (Ahmad et al., 1998; Zhu et al., 2017).

So based on these studies it is possible that in *H. volcanii*, the deregulation of the tetrahydrofolate pathway and downstream branches by either deletion of *hdrB*, the misuse of non-required *hdrb* marker on the plasmid or the under or over supplying of thymidine could have major effects on *H. volcanii* nucleotide metabolism inducing growth and cell shape deregulation. Additionally, the misregulation of the dihydrofolate reductase expression could have a direct impact on *H. volcanii* DNA replication. So far the existence of a clear cell cycle in *H. volcanii* and the coupling of DNA replication, cell division and chromosome segregation mechanisms are unknown but a potential link between replication, growth and therefore cell shape is not excluded.

Our current study does not allow for disentangling the origin of the phenotypes observed in Δ*hdrB* mutant, but it does show that the *hdrB* deletion background is not suitable for experiments about shape and that the use of non-required *hdrb* selection marker on a plasmid should be strictly avoided. pTA1392 and pTA962 are widely used in the field and this even in non Δ*hdrB* backgrounds (see examples in Supplementary Table S2) so we hope our study would convince the community of changing this practice. This would necessitate the community to develop more vectors free from the *hdrb* selection marker. However, our results show an intricate link between *H. volcanii* physiology and metabolism with cell shape that would be interesting to investigate further. In addition, the narrow period of rod development seen in batch culture seems to be sensitive to the constant availability of fresh media. Indeed, *H. volcanii* grown in the microfluidics system CellASIC ONIX remain rod, suggesting that the maintenance of rod is sensitive to either the impoverishment or the accumulation of a compound that could trigger the transition to plate shape in batch culture (de Silva et al., 2021).

As of why the sole presence of a plasmid is inducing rod formation, our study could not give an answer, but it is probable that the presence of the plasmid is a burden by itself changing the cell metabolism and affecting cell size and shape. All the studies referred to and done in this work are based upon plasmids containing the *H. volcanii* endogenous pHV2 plasmid origin (Charlebois et al., 1987; Wendoloski et al., 2001; Hartman et al., 2010). Proven to be a useful genetic tool, we however lack insights into the molecular mechanism of pHV2 origin.

As an alternative and for studies where cell shape is critical, we would highly recommend avoiding using a plasmid and favor the genomic integration for complementation or for expression of fluorescently tagged proteins for example. Gene deletions could be made by the replacement with the selection marker to restore the cells prototrophy without the necessity of a plasmidic marker. Additionally, the expression level of the selection cassette into the chromosome might resemble better the endogenous gene expression, or other promoters could be tested to fine tune the appropriate level of expression of the different selection markers to minimize impacts on cell shape. However, a plasmid free system would require the development of a genetic system that allows for the excision of the selection cassette from the genome in case of the construction of multi locus mutant strains or complementation experiments for example. A strategy that would be worth developing to move the field forward.

Aside from considerations about genetic backgrounds and plasmid usage, we would like to highlight that our study was conducted in Hv-CA medium that was not supplemented by trace elements. Previous studies (Duggin et al., 2015; de Silva et al., 2021) showed that the addition of trace elements is crucial both in Hv-CA and Hv-YPC for the proper development of cell shape. To the contrary we observe regular cell shape in Hv-CA without trace elements. However, it is most likely that sufficient trace elements are present in the media. This could be due to the water used for media preparation in our team that is deionized water and not from an ultrapure purification system as it is the case for our colleagues. And as shown before, some trace elements might be present in sufficient amount in media preparation reagents depending on their degree of purity (de Silva et al., 2021). In addition to the natural plasticity of *H. volcanii* cell shape, such differences could render difficult the comparison and the reproduction of results between laboratories, it is certain that the research community would beneficiate from a homogenization of culture conditions.

Overall, we hope our study raised awareness to the community about the impact of the current genetic system used in *H. volcanii* on cell shape and on the importance of cautiousness in choosing a genetic background for a given study. We believe that with common efforts, new standards can be achieved by the development of new methods such as a plasmid free system, new selection markers and more standardized culture conditions for moving the haloarchaea cell biology field forward.

## Data availability statement

The raw data supporting the conclusions of this article will be made available by the authors, without undue reservation.

## Author contributions

MP: Conceptualization, Data curation, Formal analysis, Methodology, Visualization, Writing – original draft, Writing – review & editing, Investigation, Validation. ID: Conceptualization, Funding acquisition, Supervision, Writing – review & editing. S-VA: Conceptualization, Funding acquisition, Supervision, Writing – review & editing. SI: Conceptualization, Data curation, Formal analysis, Methodology, Visualization, Writing – original draft, Writing – review & editing, Supervision, Validation, Investigation, Project administration.
