# Supplementary material for: “Influence of plasmids, selection markers and auxotrophic mutations on Haloferax volcanii cell shape plasticity”
Source: Front Microbiol. 2023 Sep 29;14:1270665. doi: 10.3389/fmicb.2023.1270665 (PMC10570808; doi:10.3389/fmicb.2023.1270665)
Supplement: Supplementary file 1 [file Presentation.PDF]

Supplementary Material

"Influence of plasmids, selection markers and auxotrophic mutations on *Haloferax volcanii* cell shape plasticity."

Megha Patro[1,2], Iain G. Duggin[3], Sonja-Verena Albers[1], Solenne Ithurbide[1,4*]

[1]Molecular Biology of Archaea, Institute of Biology II, Faculty of Biology, University of Freiburg, Freiburg, Germany

[2]Spemann Graduate School of Biology and Medicine, University of Freiburg, Freiburg, Germany

[3]The Australian Institute for Microbiology and Infection, University of Technology Sydney, Sydney, NSW 2007, Australia

[4]Present Address: Département de Microbiologie, Infectiologie et Immunologie, Université de Montréal, Montréal, Québec, Canada

***Correspondence:**
Solenne Ithurbide - solenne.ithurbide@umontreal.ca

1 Supplementary Material description

The supplementary material contains 10 supplementary Figures and 2 supplementary Tables:

1.1 List of Supplementary Figures:

Figure S1: Analysis of the morphology of *H. volcanii* DS2 at different stages of growth classified by cell shape types.

Figure S2: Analysis of the morphology of *H. volcanii* DS2, H26, H53, H98 and H729 at different stages of growth.

Figure S3: Growth curves of DS2, H26, H53, H98 and H729 strains in absence and presence of various plasmids

Figure S4: Comparison of cell circularity of strains with and without plasmids in H26, H53, H77, H729 and H98 backgrounds.

Figure S5: Heatmap of statistical significance of comparison test of DS2, H26, H53, H77, H98 and H729 strains across different measurements.

Figure S6: Heatmap of statistical significance of comparison test of in H26 background across different measurements.

Figure S7: Heatmap of statistical significance of comparison test of in H53 background across different measurements.

Figure S8: Heatmap of statistical significance of comparison test of in H77 background across different measurements.

Figure S9: Heatmap of statistical significance of comparison test of in H98 background across different measurements.

Figure S10: Heatmap of statistical significance of comparison test of in H729 background across different measurements.

1.2 List of Supplementary Tables:

Table S1: Plasmids, strains and culture conditions.

Table S2: Summary of studies in which an effect of a plasmid on *H. volcanii* cell shape has been observed.

2 Supplementary Figures

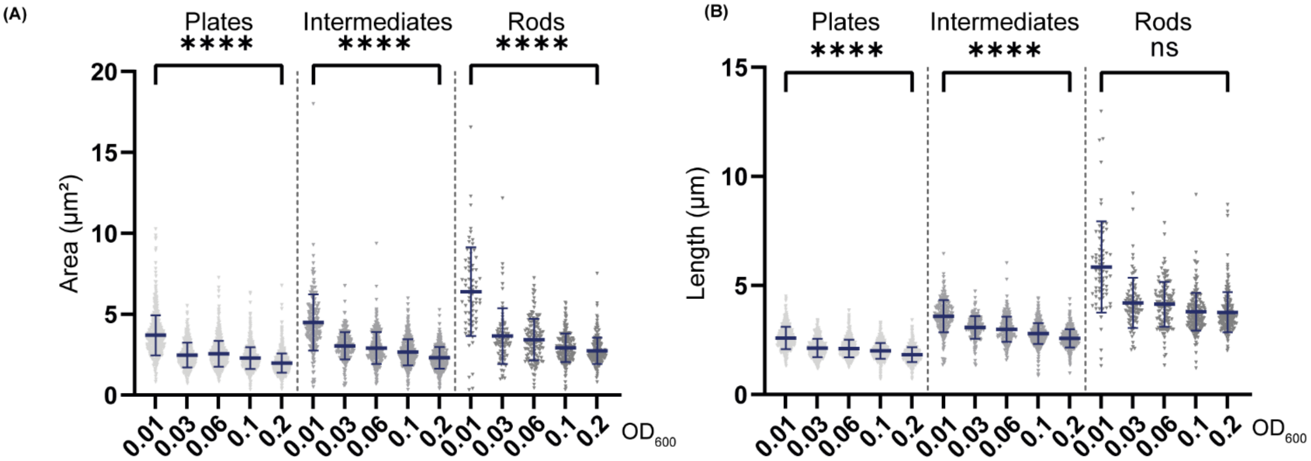

Figure S1: Analysis of the morphology of *H. volcanii* DS2 at different stages of growth classified by cell shape types. (A) Violin plot distribution of cells area (µm^2) for each cell shape types at different O.D$_{600}$ (**B**) Violin plot distribution of cells length (µm) for each cell shape types at different O.D$_{600.}$ (**A,B**) Cell types R (rods), I (intermediates) and P (plates) are determined depending on the cell circularity as described in the text. The statistical analysis were performed using Kruskal-Wallis-test in GraphPad Prism and data represent more than 1300 cells from three independent experiments. Black line indicates mean; bottom and top lines indicate the Standard deviation.

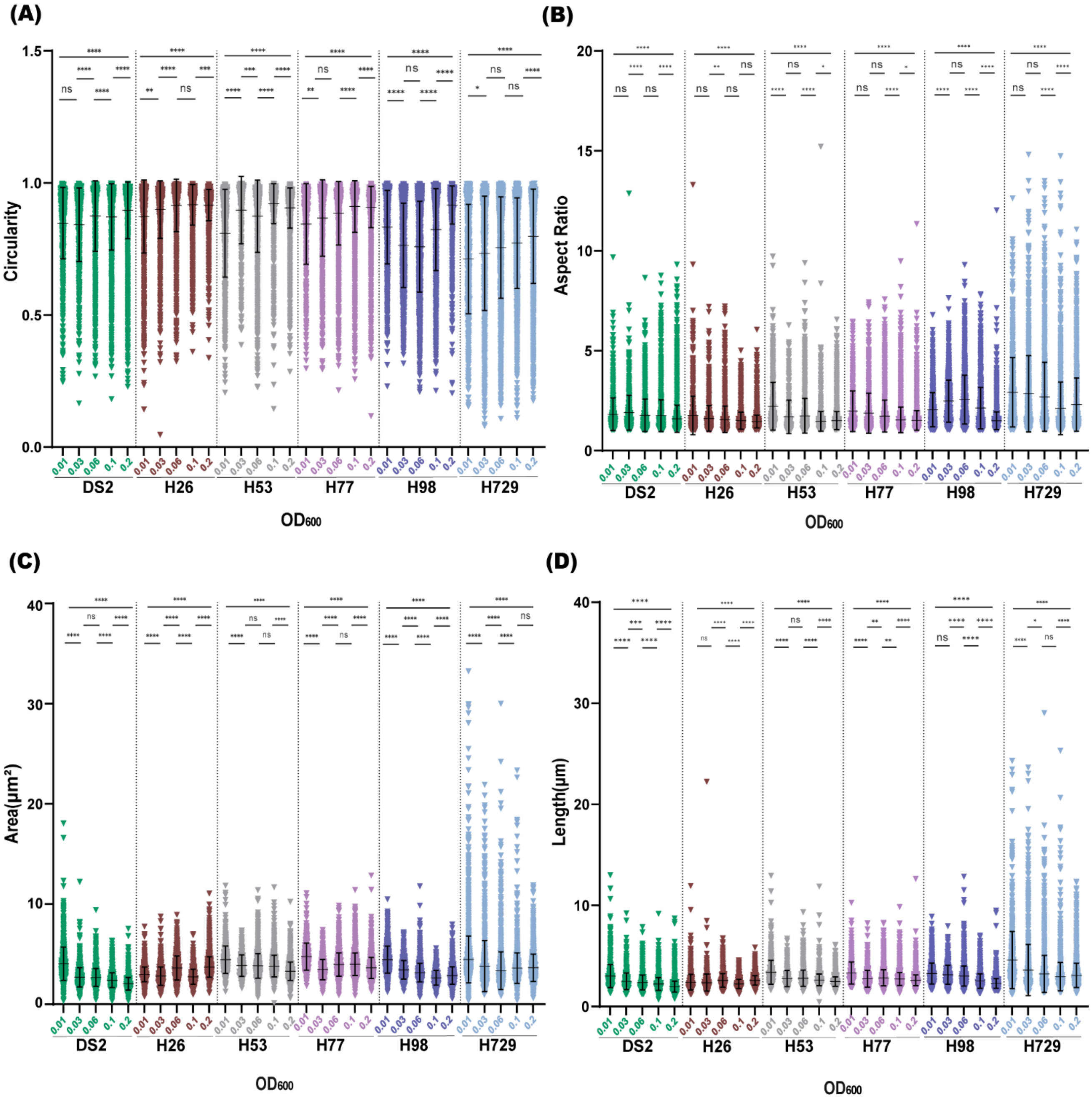

Figure S2: Analysis of the morphology of *H. volcanii* DS2, H26, H53, H77, H98 and H729 at different stages of growth.

(A) Violin plot distribution of cells circularity at different O.D$_{600.}$ Data set is the same as in Figure 2B. (**B**) Violin plot distribution of cells aspect ratio at different O.D$_{600}$ (**C**) Violin plot distribution of cells area (µm^2) at different O.D$_{600}$ (**D**) Violin plot distribution of cells length (µm) at different O.D$_{600}$. The statistical analysis in **(A, B, C, D)** were performed using Kruskal-Wallis-test in GraphPad Prism and data represent more than 500 cells from three independent experiments. Black line indicates mean; bottom and top lines indicate the Standard deviation. Additional results of Kruskal-Wallis-tests are represented in Figure S5.

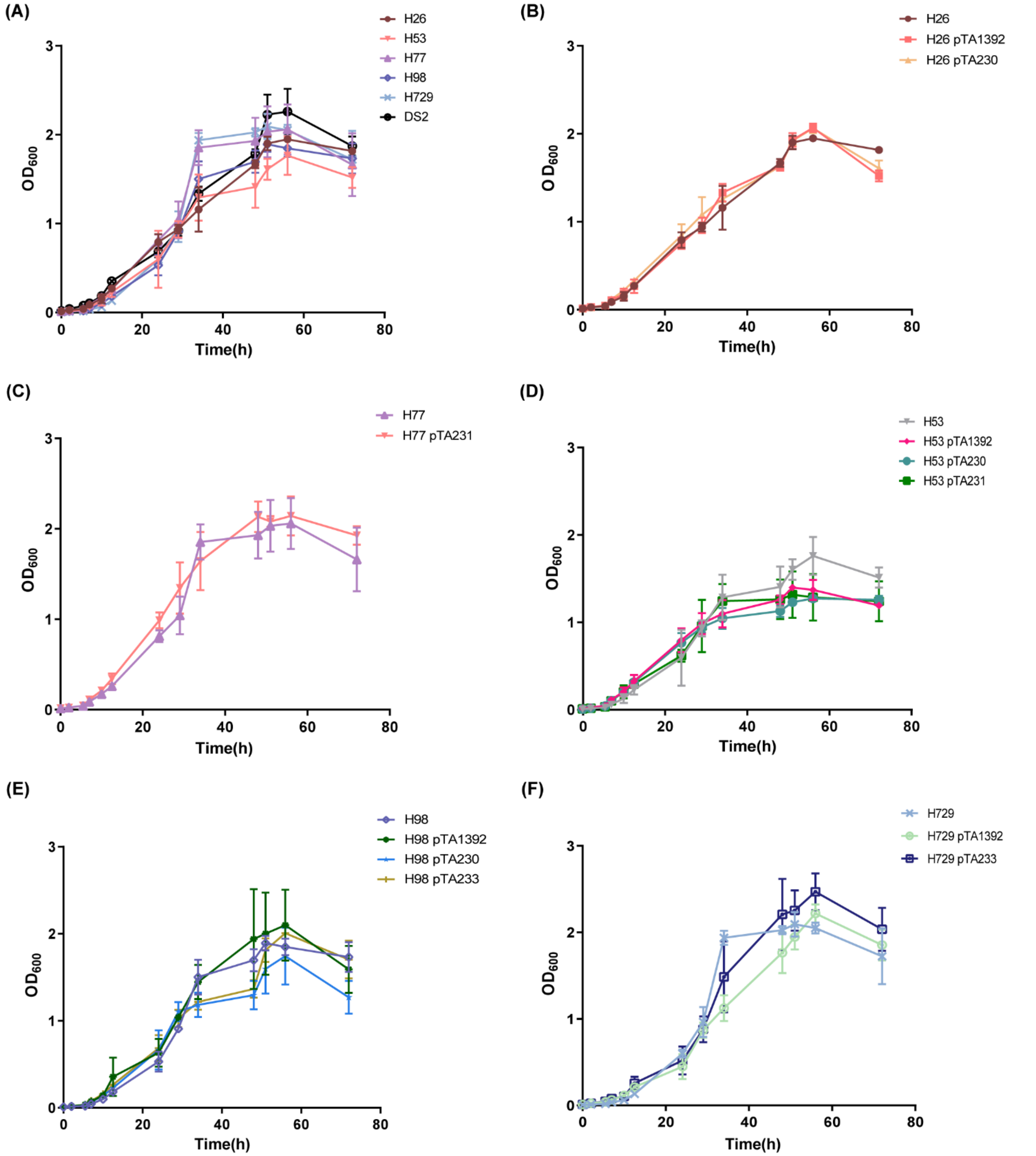

Figure S3: Growth curves of DS2, H26, H77, H53, H98 and H729 strains in absence and presence of various plasmids. Optical density (O.D_{600}) measurements of *Haloferax volcanii* strains grown in HV-CA medium supplemented with appropriate requirements (see methods and table X) at 42°C, 200 rpm. (A) Comparison of growth of DS2, H26, H53, H77, H98 and H729 without plasmids (B) Comparison of growth of H26 background strains. (**C**) Comparison of growth of H77 background strains. (D) Comparison of growth of H53 background strains. (E) Comparison of growth of H98 background strains. (F) Comparison of growth of H729 background strains. (B to F) H26 is used as comparative control and represented as dotted lines with round symbols (brown). In every graph, each curve represents the mean of three independent replicates.

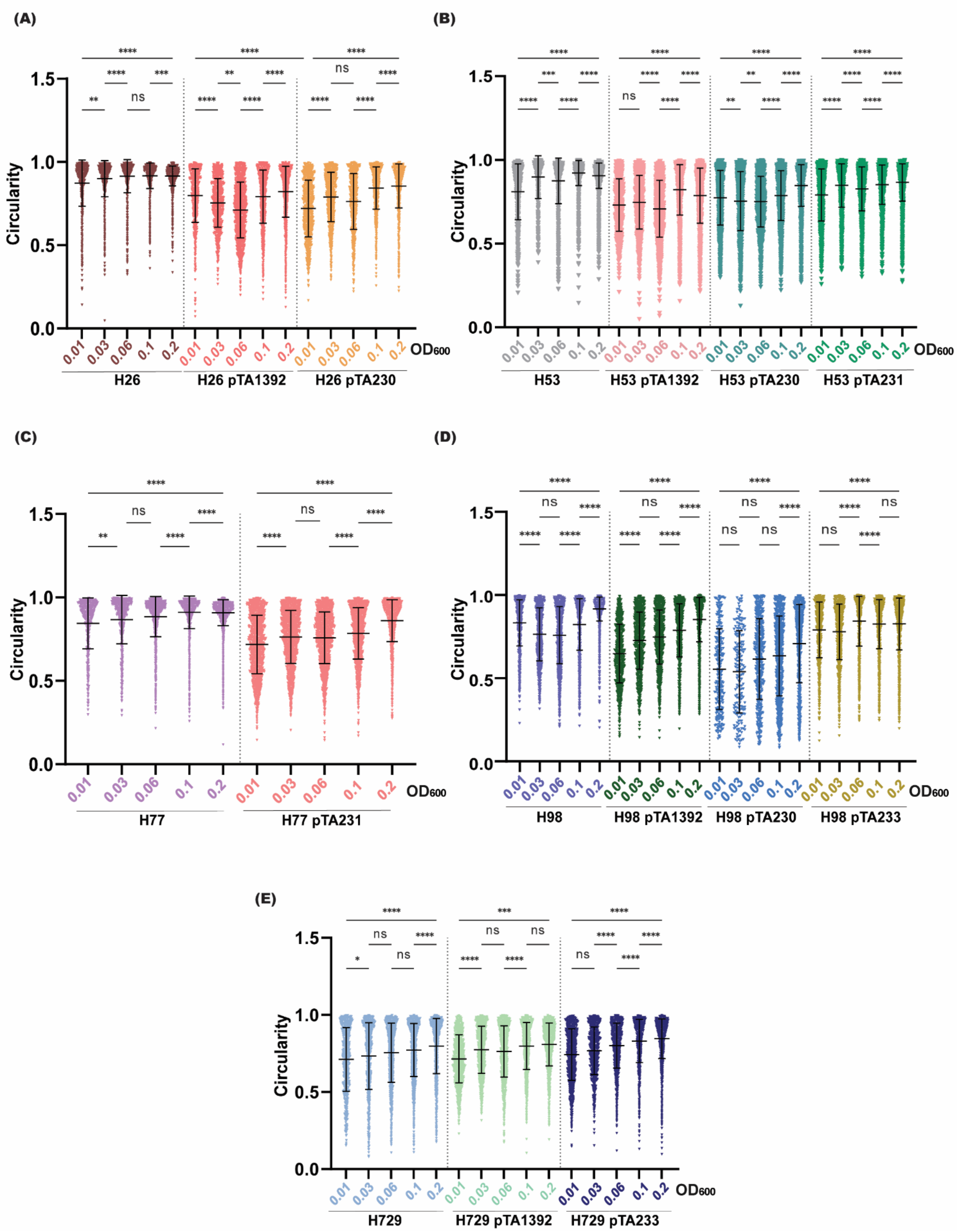

(A)
Circularity
1.5
1.0
0.5
0.0
0.01
0.03
0.06
0.1
0.2
OD600
H26
H26 pTA1392
H26 pTA230
(B)
H53
H53 pTA1392
H53 pTA230
H53 pTA231
(C)
H77
H77 pTA231
(D)
H98
H98 pTA1392
H98 pTA230
H98 pTA233
(E)
H729
H729 pTA1392
H729 pTA233

Figure S4: Comparison of cell circularity of strains with and without plasmids in H26, H53, H77, H729 and H98 backgrounds.

Violin plot distribution of cells circularity at different $O.D_{600}$ in (**A**) H26 background, (**B**) H53 background, (**C**) H77 background, (**D**) H729 background, and (**E**) H98 background. The statistical analysis in (**A**, **B, C, D, E**) were performed using Kruskal-Wallis-test in GraphPad Prism and each data represents more than 500 cells from three independent experiments. Black line indicates mean; bottom and top lines indicate the standard deviation. Additional results of Kruskal-Wallis-tests are represented in Figure S6 to S10.

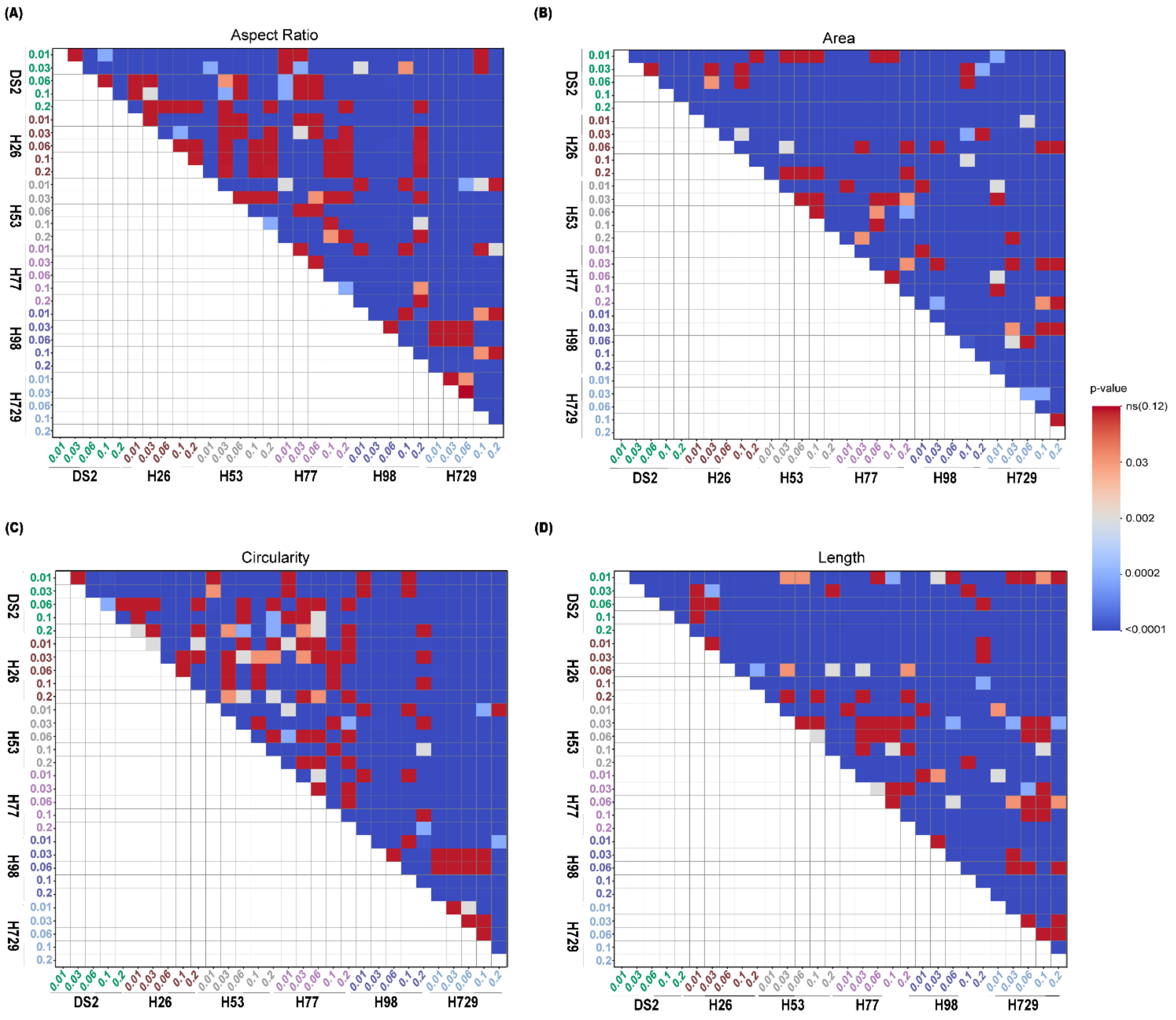

Figure S5: Heatmap of statistical significance of comparison test of DS2, H26, H53, H77, H98 and H729 strains across different measurements.

The heatmap illustrates the statistical significance from different experimental measurements (**A**) Aspect ratio, (**B**) Area, (**C**) Circularity, and (**D**) Length. The statistical significance was determined using non-parametric ANOVA Kruskal-Wallis-tests. The colour scale indicates the level of significance: Red (ns), Orange (p-value < 0.03, *), Grey (p-value < 0.002, **), Light blue (p-value < 0.0002, ***), Blue (p-value < 0.0001, ****).

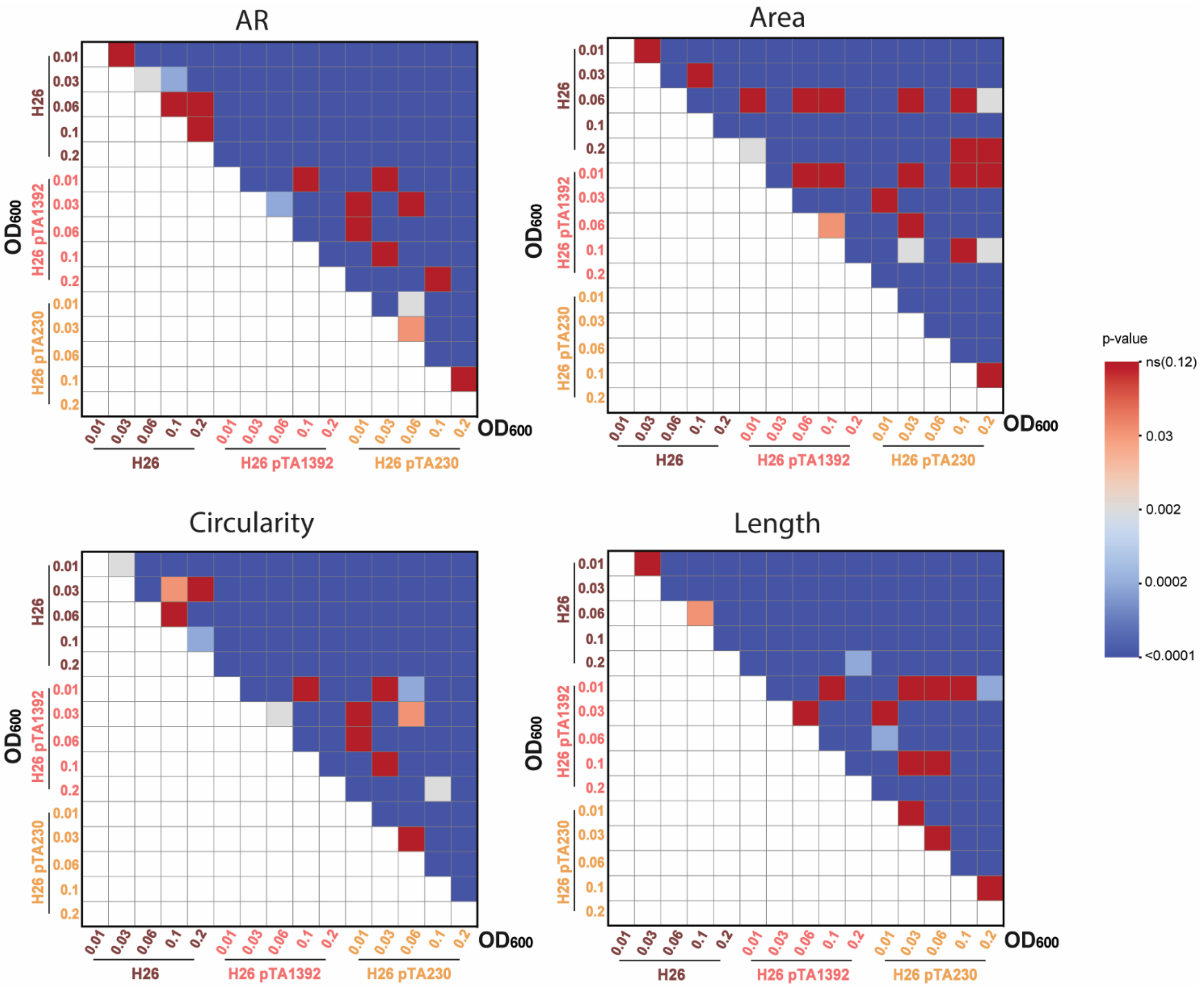

Figure S6: Heatmap of statistical significance of comparison test of in H26 background across different measurements.

The heatmap illustrates the statistical significance from different experimental measurements (**A**) Aspect ratio, (**B**) Area, (**C**) Circularity, and (**D**) Length The statistical significance was determined using non-parametric ANOVA Kruskal-Wallis-tests. The colour scale indicates the level of significance: Red (ns), Orange (p-value < 0.03, *), Grey (p-value < 0.002, **), Light blue (p-value < 0.0002, ***), Blue (p-value < 0.0001, ****).

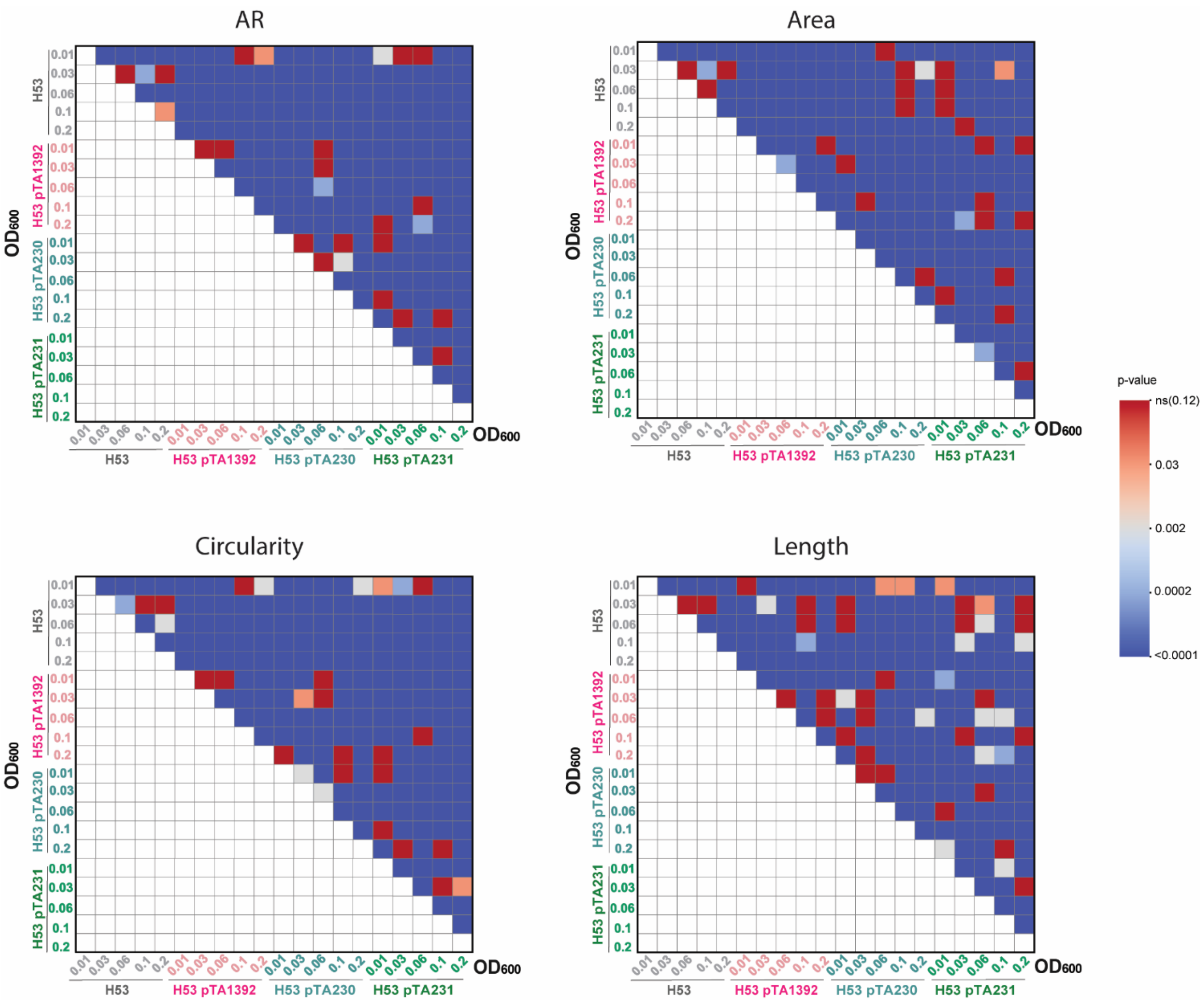

Figure S7: Heatmap of statistical significance of comparison test of in H53 background across different measurements.

The heatmap illustrates the statistical significance from different experimental measurements (**A**) Aspect ratio, (**B**) Area, (**C**) Circularity, and (**D**) Length The statistical significance was determined using non-parametric ANOVA Kruskal-Wallis-tests. The colour scale indicates the level of significance: Red (ns), Orange (p-value < 0.03, *), Grey (p-value < 0.002, **), Light blue (p-value < 0.0002, ***), Blue (p-value < 0.0001, ****).

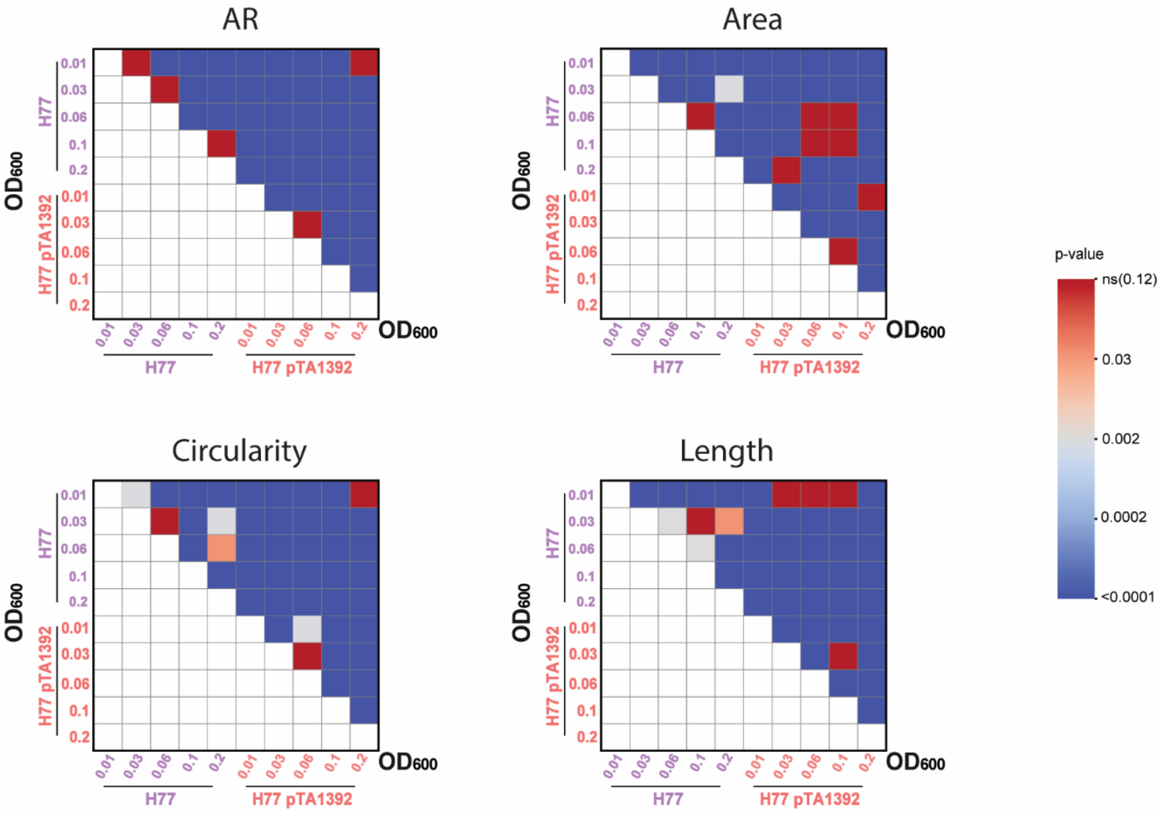

Figure S8: Heatmap of statistical significance of comparison test of in H77 background across different measurements.

The heatmap illustrates the statistical significance from different experimental measurements (**A**) Aspect ratio, (**B**) Area, (**C**) Circularity, and (**D**) Length The statistical significance was determined using non-parametric ANOVA Kruskal-Wallis-tests. The colour scale indicates the level of significance: Red (ns), Orange (p-value < 0.03, *), Grey (p-value < 0.002, **), Light blue (p-value < 0.0002, ***), Blue (p-value < 0.0001, ****).

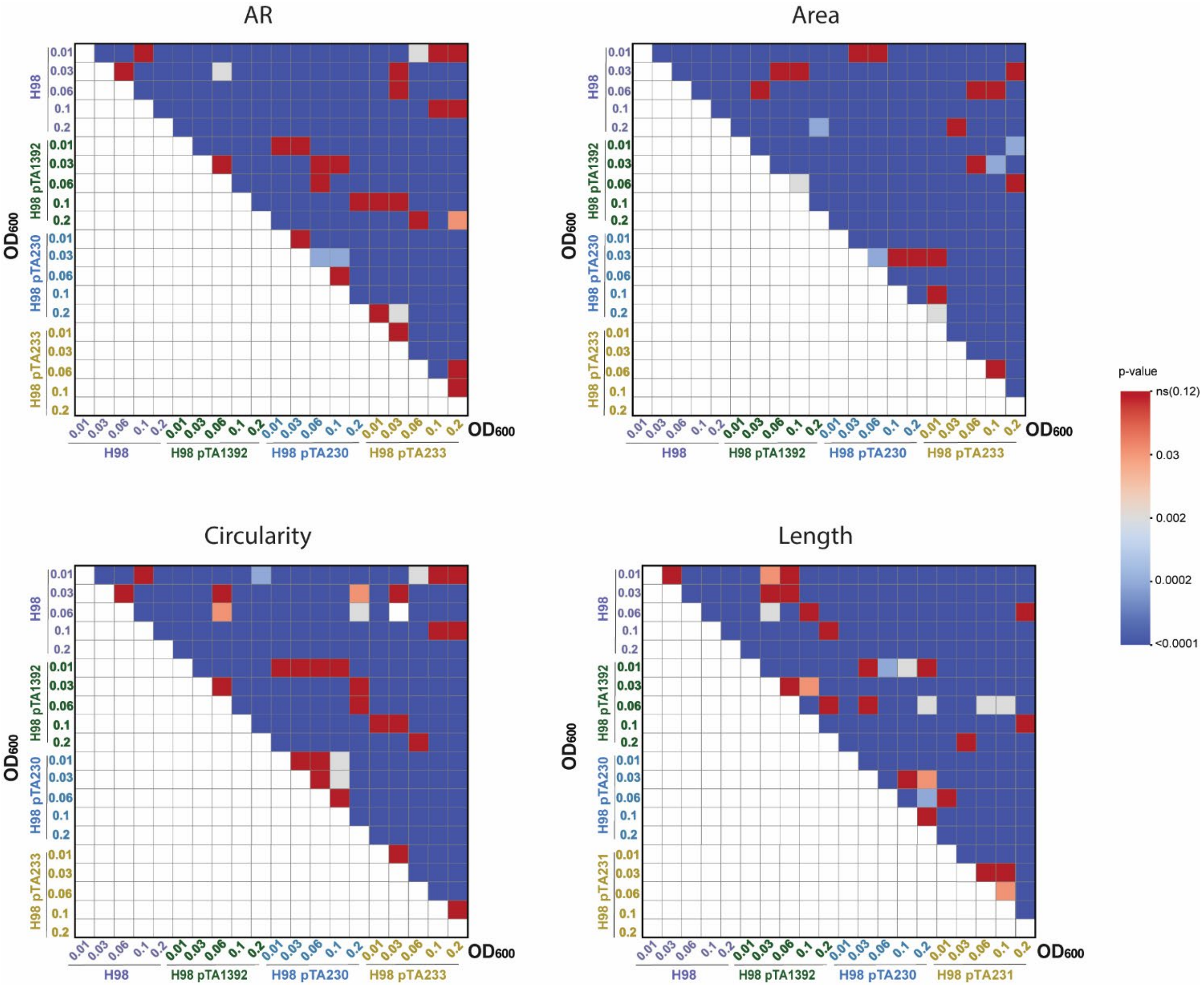

Figure S9: Heatmap of statistical significance of comparison test of in H98 background across different measurements.

The heatmap illustrates the statistical significance from different experimental measurements (**A**) Aspect ratio, (**B**) Area, (**C**) Circularity, and (**D**) Length The statistical significance was determined using non-parametric ANOVA Kruskal-Wallis-tests. The colour scale indicates the level of significance: Red (ns), Orange (p-value < 0.03, *), Grey (p-value < 0.002, **), Light blue (p-value < 0.0002, ***), Blue (p-value < 0.0001, ****).

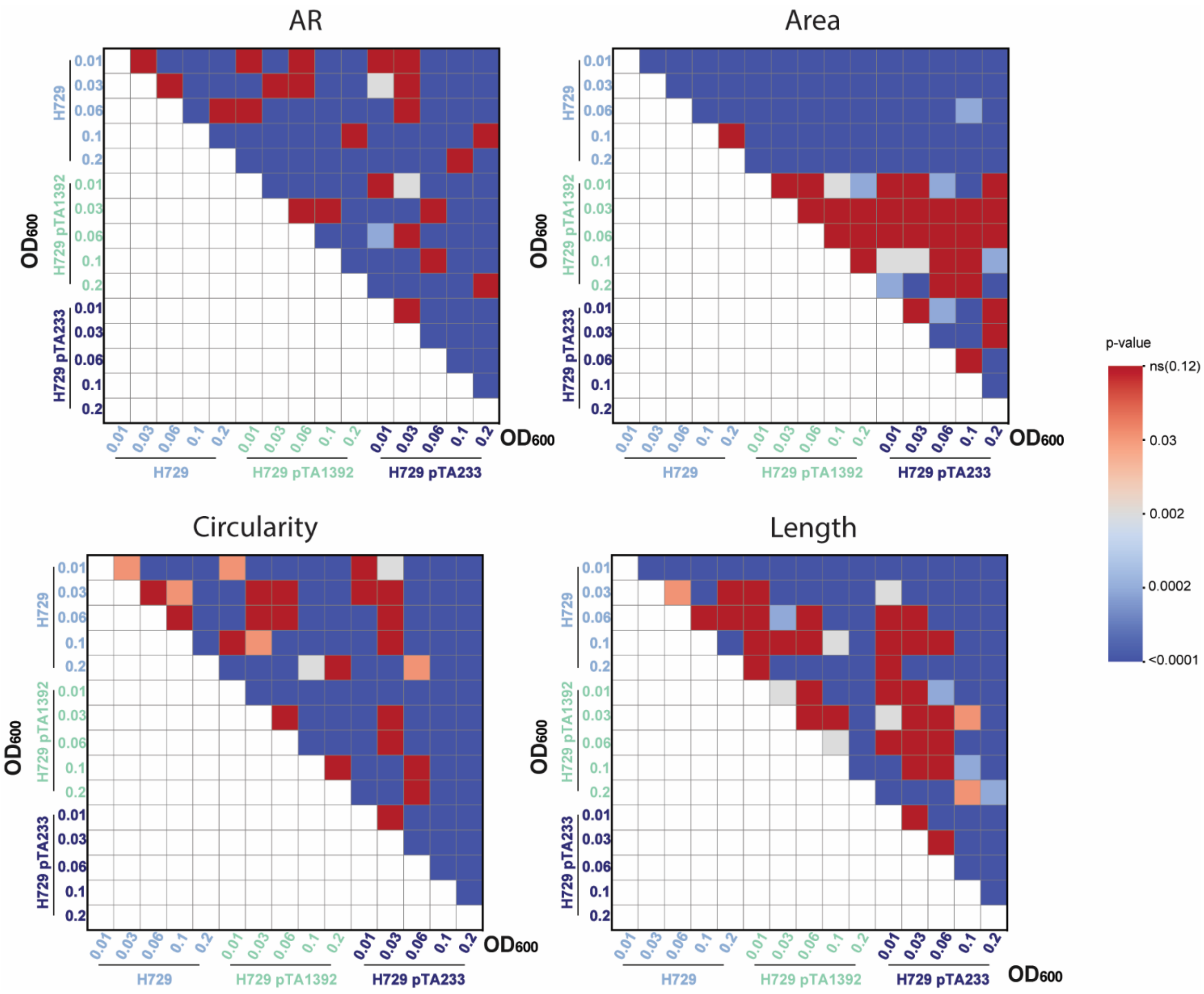

Figure S10: Heatmap of statistical significance of comparison test of in H729 background across different measurements.

The heatmap illustrates the statistical significance from different experimental measurements (**A**) Aspect ratio, (**B**) Area, (**C**) Circularity, and (**D**) Length The statistical significance was determined using non-parametric ANOVA Kruskal-Wallis-tests. The colour scale indicates the level of significance: Red (ns), Orange (p-value < 0.03, *), Grey (p-value < 0.002, **), Light blue (p-value < 0.0002, ***), Blue (p-value < 0.0001, ****).

3 Supplementary Tables

Table S1 (Part I): Plasmids, strains and culture conditions.

1 **1. Plasmids used in this study**		
**Plasmid**	**Properties**	**Reference**
pTA1392	Derived from pTA131* containing pHV2 origin with *pyrE2* and *hdrB* markers under control of the constitutive promoter P*fdx*, Amp^R	(Haque et al., 2020)
pTA230	Derived from pTA131* containing pHV2 origin with *pyrE2* marker under control of the constitutive promoter P*fdx*, Amp^R	(Allers et al., 2004)
pTA231	Derived from pTA131* containing pHV2 origin with *trpA* marker under control of the constitutive promoter P*fdx*, Amp^R	(Allers et al., 2004)
pTA233	Derived from pTA131* containing pHV2 origin with *hdrB* marker under control of the constitutive promoter P*fdx*, Amp^R	(Allers et al., 2004)
*pTA131: pBluescript II derived plasmid containing pyrE2 under the ferredoxin constitutive promoter Pfdx-pyrE2 (Allers et al., 2004)		

**2. *H. volcanii* background strains and culture conditions used in this study**				
**Strain**	**Genotype**	**Additives for growth**		**Reference**
		Hv-YPC	Hv-Ca	
DS2	WT	-	-	(Mullakhanbhai and Larsen, 1975)
H26	(DS70**) Δ*pyrE2*	-	+ Ura	(Allers et al., 2004)
H53	(DS70**) Δ*pyrE2* Δ*trpA*	-	+ Ura + Trp	(Allers et al., 2004)
H77	(DS70**) Δ*trpA*	-	+ Trp	(Allers et al., 2004)
H98	(DS70**) Δ*pyrE2* Δ*hdrB*	+Thy	+ Ura + Thy + Hyp	(Allers et al., 2004)
H729	(DS70**) Δ*hdrB*	+Thy	+Thy + Hyp	T. Allers strain collection

Table S1 (Part II): Plasmids, strains and culture conditions.

**3.** ***H. volcanii*** **strains transformed with plasmid and culture conditions used in this study**			
**Strain**	**Strain Genotype**	**Plasmid markers**	**Additives for growth in Hv-Ca**
H26 pTA1392	(DS70**) Δ*pyrE2*	*pyrE2, hdrB*	-
H26 pTA230	(DS70**) Δ*pyrE2*	*pyrE2*	-
H53 pTA1392	(DS70**) Δ*pyrE2* Δ*trpA*	*pyrE2, hdrB*	+ Trp
H53 pTA230	(DS70**) Δ*pyrE2* Δ*trpA*	*pyrE2*	+ Trp
H53 pTA231	(DS70**) Δ*pyrE2* Δ*trpA*	*trpA*	+ Ura
H77 pTA231	(DS70**) Δ*trpA*	t*rpA*	-
H98 pTA1392	(DS70**) Δ*pyrE2* Δ*hdrB*	*pyrE2, hdrB*	-
H98 pTA230	(DS70**) Δ*pyrE2* Δ*hdrB*	*pyrE2*	+ Thy + Hyp
H98 pTA233	(DS70**) Δ*pyrE2* Δ*hdrB*	*hdrB*	+ Ura
H729 pTA1392	(DS70**) Δ*hdrB*	*pyrE2, hdrB*	-
H729 pTA233	(DS70**) Δ*hdrB*	*hdrB*	-
** DS70: Wild-type *H. volcanii* DS2 cured of pHV2 (Wendoloski et al., 2001)<br><br>Ura: 50 µg/mL uracil, Trp: 50 µg/mL tryptophan, Thy: 40 µg/mL thymidine, Hyp: 40 µg/mL hypoxanthine.			

Table S2: Summary of studies in which an effect of a plasmid on *H. volcanii* cell shape has been observed.

Strain	Strain Genotype	Plasmid	Cultivation media	General observation	Reference
H53	Δ*trp*Δ*pyrE2*	pTA963 P_{fdx}-*pyrE2-hdrB*	Hv-CA + 50 µg/ml trp	90% of rods in H53 + pTA963 compared to 10% in H53 without plasmid in cultures of colonies transferred from agar plates to liquid.	(Abdul Halim et al., 2016)
H26	Δ*pyrE2*	No plasmid	Hv-CA+ 50 µg/ml uracil	Report of the early log phase rod development	(Li et al., 2019)
H26	Δ*pyrE2*	pTA962 P_{fdx}-*pyrE2-hdrB*	Hv-YPCab (addition of trace elements to YPC)	Observation of a peak of rod cells in early culture for H26 + pTA962 whereas this is not present for H26 without plasmid.	(de Silva et al., 2021)
H98	Δ*pyrE2*Δ*hdrB*	pTA962 P_{fdx}-*pyrE2-hdrB*	Hv-YPCab (addition of trace elements) and other media	Study of the impact of growth conditions on the early culture rod formation. All the strains carry a plasmid.	
H53	Δ*trp*Δ*pyrE2*	pTA963 P_{fdx}-*pyrE2-hdrB*	Hv-CA + 50 µg/ml trp	Corroborate the results from Abdul Halim et al., 2016. Rod cells are observed in early log phase.	
ID77	(H98 Δ*pyrE2*Δ*hdrB*) p.fdx-hdrB ΔftsZ2	pTA962 P_{fdx}-*pyrE2-hdrB*	Hv-Cab (contains vitamins biotin and thiamine and trace elements)	Difference of cell shape between (H98) p.fdx-hdrB ΔftsZ2 strain (giant dimorphic plate) VS (H98) p.fdx-hdrB ΔftsZ2/pTA962 (filamentuous rods)	(Liao et al., 2021)
HTQ 239	(H98 Δ*pyrE2*Δ*hdrB*) p.fdx-hdrB sepF ::ptnaA_sep F	pTA962 P_{fdx}-*pyrE2-hdrB*	Hv-Ca +vitamins biotin and thiamine	SepF depletion strain are giant amorphic plate cells whereas the presence of pTA1392 render the cells filamentous	(Nußbaum et al., 2021)
